# Expression of cerebral serotonin related to anxiety-like behaviors in C57BL/6 offspring induced by repeated subcutaneous prenatal exposure to low-dose lipopolysaccharide

**DOI:** 10.1371/journal.pone.0179970

**Published:** 2017-06-26

**Authors:** Pei-Tan Hsueh, Hsuan-Han Wang, Chiu-Lin Liu, Wei-Fen Ni, Ya-Lei Chen, Jong-Kang Liu

**Affiliations:** 1 Department of Biological Sciences, National Sun Yat-sen University, Kaohsiung, Taiwan; 2 Department of Biotechnology, National Kaohsiung Normal University, Kaohsiung, Taiwan; Max Delbruck Centrum fur Molekulare Medizin Berlin Buch, GERMANY

## Abstract

Prenatal exposure to lipopolysaccharide (LPS), which likely occurs due to infection or contact with environmental allergens during pregnancy, is a proposed risk factor that induces anxiety- and autism spectrum disorder-like behaviors in offspring. However, the molecular and behavioral changes in offspring after maternal immune activation have not been completely identified. We hypothesized that a subcutaneous injection of LPS in a pregnant mouse would induce changes in cerebral serotonin (5-HT) in parallel to the appearance of anxiety-like behaviors in the dam’s offspring. After LPS injections (total, 100 μg/Kg), the time spent in the central region during the open field test and the number of times that the mice moved between the light and dark boxes and between the open and closed arms on the elevated plus maze test revealed anxiety-like behaviors in offspring at 5, 6 and 9 weeks of age. The mRNA expression levels of tph2 (5-HT synthesizing enzyme) and slc6a4 (5-HT transporter) were down-regulated in both adolescent (5 weeks of age) and adult (8 weeks of age) brains. Immunohistochemistry revealed that the numbers and sizes of tph2-expressing cells were notably decreased in the raphe nuclei of the midbrain of adults. Moreover, compared with controls (phosphate-buffered saline-treated offspring), the cerebral 5-HT concentration at adolescence and adulthood in LPS-induced offspring was significantly decreased. We concluded that maternal immune activation induced by exposure to a low dose of LPS decreased cerebral 5-HT levels in parallel to the down-regulation of the tph2 and slc6a4 genes and in conjunction with anxiety-like behaviors in offspring.

## Introduction

Autism spectrum disorder (ASD), a neurodevelopmental disorder, encompasses social and communication impairment and ritualistic repetitive behaviors in combination with various degrees (from extreme mild to severity) of hyperactivity, intellectual disability and anxiety [1]. Particularly, children with ASD often have comorbid traditional or atypical anxiety disorders [2]. The etiology of ASD is complicated and includes multiple genetic defects, maternal diabetes, autoimmunity preeclampsia, inflammation, exposure to pollutants and drug abuse during pregnancy, as well as unknown factors [3]. However, the underlying molecular and cellular mechanisms of anxiety disorders or ASD are largely unknown. A variety of neurotransmitters are involved in anxiety under normal conditions but appear to be imbalanced in the pathophysiology of anxiety- and ASD-like disorders. The depletion of serotonin (5-HT) concentration and defects of the 5-HT synthesizing enzyme (tph2) in the brain, hyperserotonemia, the time course of 5-HT release and variants of the different types of 5-HT receptors (htr1a, htr1d, htr2a, htr5a) and solute transporters (slc6a4) have been linked to anxiety or ASD-like behaviors [4–8]. Mesolimbic and mesocortical dopamine (DA) systems are believed to mediate various emotions, including fear and anxiety [9]. In Sprague-Dawley rats with DA depletion caused by bilateral DA lesion surgeries, anxiety- and depression-like behaviors appeared while the rats recovered normal behaviors after treatment with L-DOPA, a precursor of DA [10]. Interestingly, 5-HTergic terminals can uptake and convert exogenous L-DOPA to DA, but this process can also potentially lead to oxidative damage to the brain [11, 12]. Specific 5-HT receptor subtypes (htr1b, htr1d and htr6) have been suggested to manipulate mesolimbic DA transmission [13, 14]. Changes in the plasma or serum concentrations and genetic variants of synthesizing enzymes, receptors or transporters in both the 5-HT and DA systems have been reported to co-occur in psychiatric disorders. For example, cerebral monoamine oxidase activity that catalyzes the metabolism of monoamine neurotransmitters, such as 5-HT and DA, was significantly lower by 20.6% in autistic groups compared with that in normal controls [15].

Lipopolysaccharide (LPS), a cell wall component of gram-negative bacteria, is known as an immunostimulant that elicits production of systemic inflammatory cytokines and chemokines, expansion of leukocytes in bone marrow, activation of the complement cascade, activation of the hypothalamic-pituitary adrenal (HPA) axis and enhancement of 5-HTergic and DAergic activity in the cerebrum [16–18]. In rodents, maternal immune activation through bacterial infection or exposure to allergens, usually administration of LPS, during pregnancy can produce long-lasting anxiety- and ASD-like behaviors in offspring. However, the specific long-lasting effects of prenatal LPS exposure on physiological and behavioral characteristics have been unclear due to the different animal strains, stimulation time, doses and administrative routes used in the various studies [19–22]. For example, exposing maternal C57BL/6 mice to 120 μg/Kg LPS by intraperitoneal (ip) injection improved the anxiety-like behaviors of their offspring on the elevated plus maze (EPM) assay [22]. Depino et al. have further reported that prenatal exposure to 25 μg/Kg LPS (ip) during pregnancy induced anxiety-like behaviors in male offspring on the EPM, light-dark box (LDB) and open-field test (OFT) at 8–10 weeks of age [23]. However, Xuan et al. did not observe any significant differences in thigmotaxis (an indicator of murine anxiety-like behavior in the OFT) in the offspring of the maternal immune activation (ip, total, 150 μg/Kg) or saline control groups [20]. Babri et al. has reported that prenatal exposure to LPS (ip, 500 μg/Kg) did not alter anxiety-like behaviors of C57BL/6 offspring in the OFT, EPM and LDB tests [21].

In this study, we aimed to analyze the long-lasting effects of maternal immune activation on offspring. We hypothesized that the induction of maternal immune activation in C57BL/6jNar1 (C57BL/6) mice with continuous subcutaneous (sc) injections of a low dose of LPS during the gestation period (designed to mimic mild bacterial infections or repeated exposure to environmental allergens during pregnancy) would result in changes in anxiety-like behaviors and anxiety-related neurotransmitters, including 5-HT and DA, in the cerebrum of the offspring.

## Materials and methods

### Animal care

All animal experiments in this study were conducted in accordance with the Animal Protection Act (Taiwan) and the Guide for the Care and Use of Laboratory Animals (National Animal Laboratory Center, Taiwan). All animal handling protocols were approved by the Institutional Animal Care and Use Committee of the National Kaohsiung Normal University, Taiwan (approval ID: 10601).

C57BL/6 mice were purchased from the National Laboratory Animal Center (NLAC, Taipei, Taiwan) and bred under non-SPF (specific-pathogen free) conditions with a strict 12-h light/dark cycle at 22–24°C and 55–65% humidity. To obtain offspring, at the age of 8 weeks, two female mice (ca. 20 g) were paired with one male mouse (ca. 24 g). The mice were separated after the appearance of vaginal plugs in the females. The mating day was recognized as gestational day (GD) 0. No mice that previously mated were used in this study. The pregnant mice were individually housed. When the offspring were 4 weeks old, they were separated from the dams into cages with 1–5 female offspring/cage. All mice were provided access to a standard food (MFG; Oriental Yeast Co., Chiba, Japan).

### Experimental design

**Fig 1:**
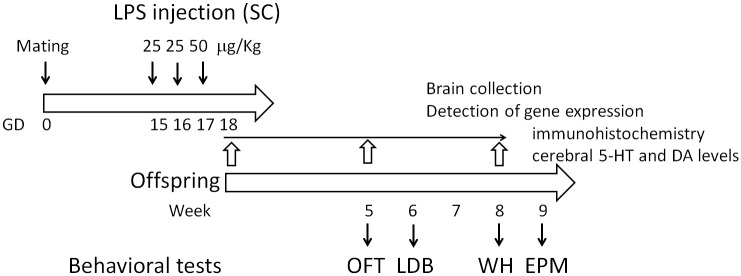
Experimental design. On gestation day (GD) 15, 16 and 17, pregnant mice were subcutaneously injected with 25, 25 and 50 μg/Kg of LPS, respectively. Pregnant control mice were injected with phosphate-buffered saline (PBS). The open field test (OFT), light/dark box (LDB), wire hanging (WH) and elevated plus maze (EPM) behavioral tests were conducted when the offspring were 5 weeks old, 6 weeks old, 8 weeks old and 9 weeks old, respectively. Brains were dissected from fetuses on GD18, adolescent mice at 5 weeks old and adult mice at 8 weeks old. mRNA expression, immunohistochemistry and neurotransmitter levels (5-HT [serotonin] and DA [dopamine]) were examined.

The dose effects of LPS on abortion in pregnant mice and the survival of the offspring were preliminarily evaluated (S1 Table). Abortions occurred in approximately 60–66% of the pregnant mice injected (sc) with *E*. *coli* O55:B5 LPS (Sigma, St. Louis, MO, USA) at doses of 50–60 μg/Kg on GD15, 50–60 μg/Kg on GD16 and 50–80 μg/Kg on GD17, whereas abortions occurred in less than 11% of the mice totally injected with 100 μg/Kg of LPS (S1 Table). Thus, in this study, maternal immune activation was induced using the following LPS injection (sc) conditions: 25 μg/Kg on GD15, 25 μg/Kg on GD16 and 50 μg/Kg on GD17. As controls, pregnant mice were injected (sc) with 500 μL phosphate-buffered saline (PBS) on GD15, GD16 and GD17. To avoid the interferences of gender effect on anxiety, only female mice were used in this study. After 4 weeks of age, the body weights of the PBS-treated controls (n = 30) and LPS-induced (n = 30) offspring were recorded every week. The timeline of the brain tissues extraction and the behavioral tests of the offspring is shown in Fig 1.

### Open-field test (OFT)

At the age of 5 weeks, the offspring (n = 30, each group) were subjected to the OFT; the apparatus consists of an area of 60 x 60 cm with 36 squares (10 x 10 cm per square; outer, 20 squares; inner, 16 squares) with illumination of 65 lux. Initially, a single offspring was placed in the central area and allowed to move freely for 10 min. The total distance traveled and the amount of time spent in the central area were recorded by a video camera and analyzed by Tracker software (version 4.96; open source: http://physlets.org/tracker/)

### Light/Dark box (LDB) test

The LDB test was performed when the offspring were 6 weeks old (n = 30, each group). The LDB apparatus consists of a light box (45 x 27 x 45 cm; 45 lux) and dark box (45 × 27 x 45 cm; <1 lux), which are joined by a wall containing an open door (7.5 x 7.5 cm). The offspring were placed in the central illuminated area facing the wall and were allowed to move between the two boxes for 10 min. The number of times that the mice moved between the light and dark boxes was recorded.

### Wire hanging (WH) test

For the WH test, an iron wire (6 mm in diameter, 35 cm long) was horizontally stretched between two walls of an open box (35 x 30 cm), which was 50 cm above the ground with illumination of 350 lux. Eight-week-old offspring (n = 30, each group) were placed on the wire in middle of the box and allowed to use their forelimbs to sustain their body weight. Until the mice fell down or reached the end of the wire on the walls, muscle strength was recorded as 0 point (held on with forelimbs but fell from wire before 30 sec), 1 point (held on with forelimbs but fell within 31 sec-120 sec), 2 points (held on with four limbs and remained on the wire for 5–30 sec), 3 points (held on with four limbs and remained on the wire for over 30 sec), 4 points (held on with four limbs and moved toward the walls for over 5 sec) and 5 points (held on with four limbs and moved toward and reached the walls). Each mouse performed at least 3 trials per test. The data from trials in which the mice remained on the wire for over 5 sec were collected.

### Elevated plus maze (EPM) test

The EPM apparatus consisted of two open and two closed arms (open arms: 30 × 5 cm, 100 lux, without wall; closed arms: 30 × 5 cm, 90 lux, surrounded by 16-cm high white-walls, no shadow) at 50 cm above the floor. The central platform, which was located at a space between the open and closed arms, was a 5 x 5 cm square with illumination of 100 lux. At the age of 9 weeks, the mice (n = 30, each group) were placed on the central platform, facing an open arm, and allowed to explore for 10 min. The total number of times that the mice moved between the open and closed arms was recorded.

### Brain tissues

The brain tissues in both the PBS-treated controls and LPS-induced groups were dissected from fetuses at GD18 and offspring at 5 and 8 weeks old. Before sacrifice, all offspring completed the behavioral tests. If the offspring were 5 weeks old, they had to remain in the central areas less than 70 sec (LPS-induced) or over 102 sec (PBS-treated) in the OFT. At 8 weeks old, the offspring had to appear less than 22 times (LPS-induced) or over 37 times (PBS-treated) in the LDB test. The times on the OFT or LDB test were based on our results in this study (see below). After testing, the mice that satisfied our conditions were immediately sacrificed. The pregnant mice at GD18 and offspring at 5 and 8 weeks old were anesthetized using Zoltil (250 μg/g, intramuscular injection [im]; Virbac Biotech. Inc., Taipei, Taiwan) and transcardially perfused with 25 mL PBS to eliminate the 5-HT contamination from residual platelets. After perfusion, the brains were carefully dissected under an anatomic microscope to remove the olfactory blub and cerebellum. All of the brain tissues, including the cerebral cortex, hippocampus, raphe nuclei, substantia nigra, midbrain, hind brain and brain stem, were kept intact. For the RNA extraction, the dissected brains were placed into RNAlater RNA Stabilization Reagent (Qiagen, Hilden, Germany) at -80°C. The dissected brains were fixed in 4% formaldehyde for wax embedding. To detect the 5-HT and DA levels by enzyme-linked immunoassay (ELISA), the dissected brains were immediately homogenized in PBS and stored at -80°C. If high performance liquid chromatography (HPLC) was performed, the dissected brains were placed into a 0.2 N perchloric acid solution (see S1 Fig legend) [17]. All of the fetal brain tissues were confirmed as female using sex-specific amplicons (male-specific sry gene, 402 bp; specific primers, forward: TGGGACTGGTGACAATTGTC, reverse: GAGTACAGGTGTGCAGCTCT; IL-3-specific primes, 544 bp; forward: GGGACTCCAAGCTTCAATCA, reverse: TGGAGGAGGAAGAAAAGCAA). The amplification profiles were set to 33 cycles, followed by 95°C for 35 sec, 50°C for 60 sec and 72°C for 60 sec [24]. All of the fetal tissues showing the presence of the IL-3 amplicon and the absence of the sry amplicon, which indicated female tissues, were collected and used for the experiments in this study.

### RNA extraction and real-time PCR

Total RNA was extracted using TRIzol reagent (Thermo Fisher Scientific Inc., Waltham, Massachusetts, USA) according to the manufacturer’s instruction. The cDNA was synthesized using an MMLV (Moloney murine leukemia virus) reverse transcriptase kit (Promega Co., Fitchburg, Wisconsin, USA). Approximately 1.88 ng cDNA was amplified by an Agilent Brilliant III Ultra-Fast qPCR SYBR Green Master Mix kit (Agilent Technologies, Santa Clara, CA, USA). The specific primers are listed in Table 1. The tbp (TATA sequence binding protein) gene has been demonstrated to be stably expressed in the mouse brain across different stages of development and in different phases of diseases, including during LPS stimulation [25, 26]. Thus, this gene was used as a reference and internal control (two independent tests per run). PCR amplification profiles were set to 40 cycles, followed by 95°C for 5 sec and 60°C for 10 sec. At the end of each reaction, the raw data were automatically analyzed, and an amplification plot was generated, as well as a calculated Cq (quantification cycle) value (Aglient AriaMx 1.0). Each gene (independent samples, n = 3) was first normalized to the reference gene using the following equation: ΔCq = Cq target gene−Cq_tbp reference gene_. To compare the PBS-treated controls and LPS-induced offspring, the fold differences in gene expression were calculated as follows: ΔΔCq = (Cq_target gene in LPS-induced group_−Cq_tbp reference gene in LPS-induced group_)–(Cq_target gene in PBS-treated control_−Cq_tbp reference gene in PBS-treated control_), and the relative fold = 0.5^ΔΔCq^ [27].

**Table 1:** Primers used in this study.

Gene	Forward (5’->3’)	Reverse (5’->3’)	Size (bp)	Accession No.	Reference
Enzyme genes					
th	AAAATCCACCAC TTAGAGACC	TAGCCACAGTACCGT CC	467	NM_009377	[65]
tph1	ATGTCTCTCGCCTCTCTCCA	TTTCCCTCAGCAGGTTCCAG	106	NM_009414	This study
tph2	ACCTGCGCAGTGATTTGAACA	CCCCAAGAGCTCATGCTGACA	108	NM_173391	This study
Receptor genes					
drd1a	ATGGCAGAGGCTTTCCCC	GCATATGCTCTTCCTCGACA	116	NM_010076	This study
htr1d	CTCTGAGACCCGGGTTGATT	GATGCTGTCAGTCCTGTCTCC	152	NM_001285482	This study
gabrg3	TCTTTCCACTGTCCCCAGAC	CAGTACTTCCCAAGCTGCTGA	105	NM_008074	This study
Transporter genes					
slc6a3	TGTGCTACCAGGGTGGAAAT	TACATGGAGCCACTCAGCTCT	105	NM_010020	This study
slc6a4	TGAGCTCTTGTGTCTGTCCAT	ATAGTTTGTAATGGGCCCGGA	71	NM_010484	This study
slc6a5	TGCCGTGTCTCTCATTTGTCT	CAGTACTGTTCACATGAGATGCT	128	XM_006540545	This study
slc8a2	TGCATCAGACCCATTACCCAC	CAGAAACCACAGAGAAGGGACA	79	NM_148946	This study

### Immunohistochemistry (IHC)

Paraffin sections were serially cut to 1 μm-slice and prepared for IHC following a standard protocol [28]. De-paraffinized tissue on slides was continuously treated with xylene and alcohol ranging from 100% to 70% concentrations and eventually placed in PBS. To expose surface antigens on the tissue, the slides were immersed in sodium citrate (pH 6.0) for NeuN (hexaribonucleotide binding protein-3, a neuronal marker), GFAP (glial fibrillary acidic protein, an astrocyte marker) and Iba1 (ionized calcium binding adaptor molecule 1, a microglial marker) immunostaining or in Tris-EDTA (ethylenediaminetetraacetic acid; pH 9.0) for tph2 (tryptophan hydroxylase 2, a synthesizing enzyme for 5-HT) and th (tyrosine hydroxylase, a synthesizing enzyme for DA) immunostaining at 100°C for 20 min. The primary antibodies listed in Table 2 were diluted in 3% BSA (bovine serum albumin)-0.1% Tween 20 PBS. Colorimetric detection of attached antibodies was performed using an UltraVision Quanto Detection System HRP Kit (Thermo Fisher Scientific Inc.) after the addition of Quanto Substrate. The antibody/polymer conjugate was visualized by applying DAB Quanto Chromogen (Thermo Fisher Scientific) dissolved in DAB Quanto Substrate buffer (Thermo Fisher Scientific) to the tissue sections for 5 min, which were then observed with light microscopy. Images were captured using a microscope (BX41; Olympus, Tokyo, Japan) equipped with a camera (TrueChromeII, Olympus). For each specimen, the total numbers (cells/mm^3^) of NeuN-, tph2- and th-expressing neurons as well as Iba1- and GFAP-expressing glial cells were automatically calculated (ImageJ; open sources, https://imagej.net/Fiji) using 10 randomly selected microscopic fields (200 X). On average, the cell numbers (cells/mm^3^) were determined using independent brain tissues (n = 6, each group). 5-HTergic axons were categorized into ≥5 μm, 2–4 μm and ≤1 μm diameters at the hillock. The proportions (%) of 5-HTergic axons in each category were calculated using 100 neurons for each sample. The resultant proportions from 6 independent tissues were averaged for each group. Quantitative evaluation of IHC images was performed using the ImageJ software. The optical densities (OD) were calculated as follows: OD_c_ = -log_10_(I_C_/I_0,C_) = A*c_C_ (I, transmitted light; I_C_, the intensity of the detected light after passing through the specimen; I_0,C_: the intensity of the light entering the specimen; A, the amount of stain with an absorption factor c) [29]. On average, the OD was calculated using independent brain tissues (n = 6, each group).

**Table 2:** Primary antibodies used for the immunohistochemistry analyses in this study.

Target	Representative to	Isotype control	Clone	Species	Dilution	Company
NeuN,	Neuron	IgG	EPR12763	rabbit	1:800	Abcam^a^
GFAP,	Astrocytes	IgG	Poly^b^	rabbit	1:2000	Abcam
Iba1,	Microglia	IgG	Poly	rabbit	1:1000	Wako^c^
th,	Dopaminergic neuron	IgG	poly	rabbit	1:800	Abcam
tph2,	Serotonergic neuron	IgG	EPR19191	rabbit	1:250	Abcam

^a^: Abcam Co., Cambridge, UK

^b^: polyclonal antibodies

^c^: Wako Co., Osaka, Japan

### Enzyme-linked immunoassay (ELISA)

The brain tissues (n = 20–26, each group), including the cerebral cortex, hippocampus, raphe nuclei, substantia nigra, midbrain, hind brain and brain stem, from the fetuses or offspring that were transcardially perfused and homogenized in 100 or 300 μL of PBS, respectively, and centrifuged at 14,000xg for 10 min. After the samples were filtered through a nylon wood membrane, the concentration of 5-HT and DA in the filtrates was determined using mouse 5-HT and DA ELISA kits (P&C Inc., Taipei, Taiwan) following the manufacturer’s instructions. No cross-reactivity to 5-hydroxyindoleacetic acid, melatonin, tryptamine or L-tryptophan in the 5-HT ELISA or adrenaline, noradrenaline, L-dopa, tyramine, homovanillic acid or tyrosine in the DA ELISA at 450 nm was observed (S1 Fig). The lower limit of detection was 0.78 ng/mL for 5-HT and 2.5 ng/mL for DA levels. Prior to testing the samples, the linearity of detection between by the ELISA and by high-pressure liquid chromatography (HPLC; the protocols are described in the legend of S1 Fig) was determined to be *r*^*2*^ = 0.8054 for 5-HT and *r*^*2*^ = 0.8412 for DA (S1 Fig).

### Statistics

The data are presented as the mean±SEM (standard error of the mean; n = 30, each behavioral test) or mean±SD (standard deviation; n = 3, qRT-PCR; n = 6, IHC examination). The one-to-one comparisons in the behavioral tests (n = 30, each behavior test), OD values and cell numbers in the IHC profiles (n = 6, independent mice) or the detection of 5-HT or DA levels (n = 22–26, 5-HT assay; n = 20–26, DA assay) was performed using the Student’s t-test. Multivariable comparison of qRT-PCR folds (n = 3, independent mice) in the cerebrum was performed using one-way ANOVA with Tukey’s post hoc test.

## Results

### Body weight, locomotive activity and muscle strength

**Fig 2:**
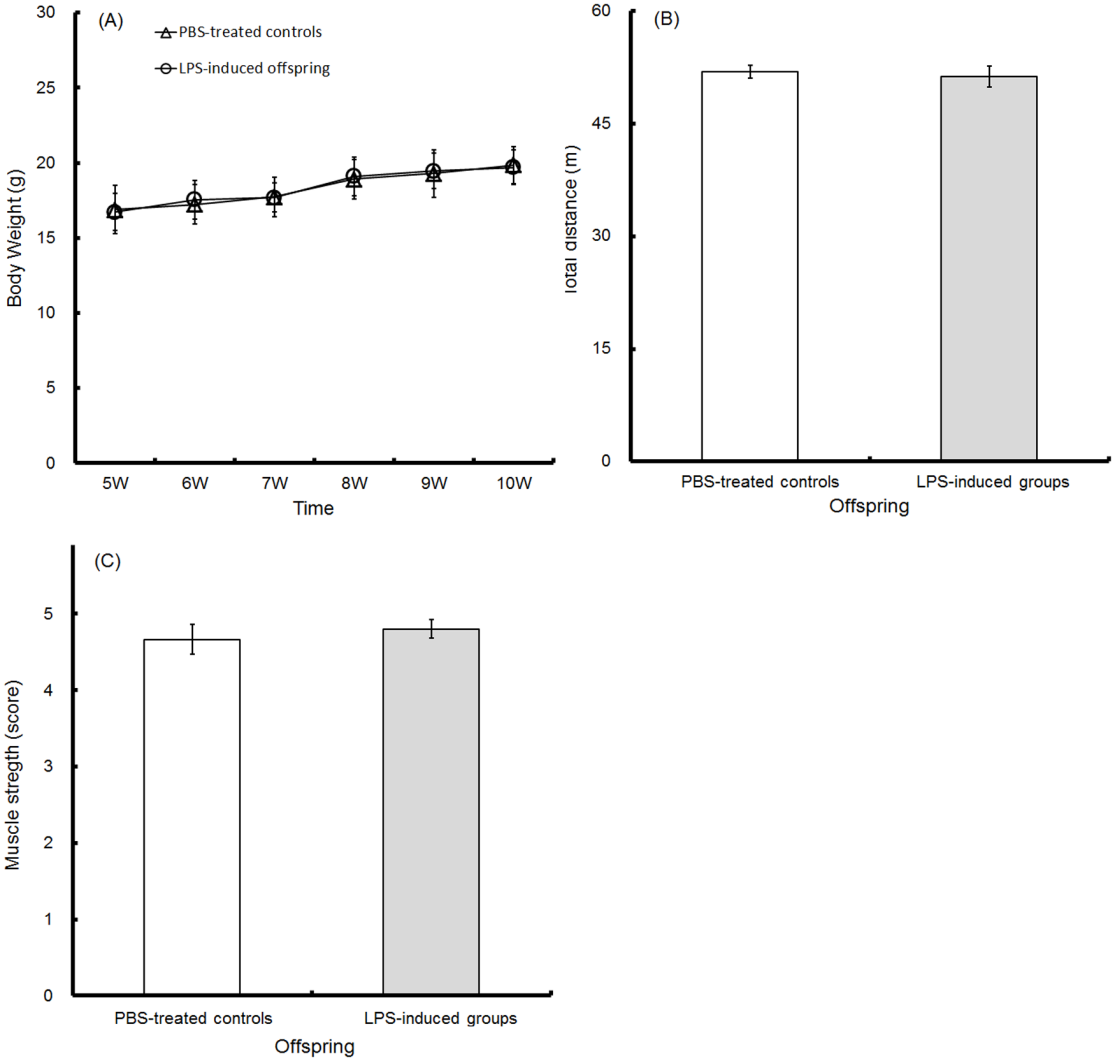
Body weight, locomotive activity and muscle strength. Mouse body weights from the ages of 5 to 8 weeks old were recorded in the PBS-treated controls (triangle, n = 30) and the LPS-induced offspring (circle, n = 30) (A). The locomotive ability and muscle strength of the PBS-treated controls (hollow, n = 30) and LPS-induced offspring (solid, n = 30) were observed at 5 and 8 weeks old, respectively. The total distance (m) of movement during the OFT (B) and the muscle strengths (scores) on the WH test (C) were recorded.

After pregnant C57BL/6 mice were injected with PBS or LPS at GD15-17, body weight, which was used as a health index for the offspring of both groups from 5 to 10 weeks old, was almost identical between the two groups (Fig 2A; t-test, p>0.5, each time point). In addition, no change occurred in the total length (m) of movement during the OFT at 5 weeks old (Fig 2B; PBS-treated controls, 51.92±0.88 m; LPS-induced offspring, 51.26±1.40 m; t-test, p = 0.69) or muscle strength as measured by the WH test at 8 weeks old (Fig 2C; PBS-treated controls, 4.67±0.19 scores; LPS-induced offspring, 4.80±0.12 scores; t-test, p = 0.57) in both groups. Overall murine health, locomotive activity and muscle strength were not significantly different between the PBS-treated controls and LPS-induced offspring.

### Anxiety-like behaviors

**Fig 3:**
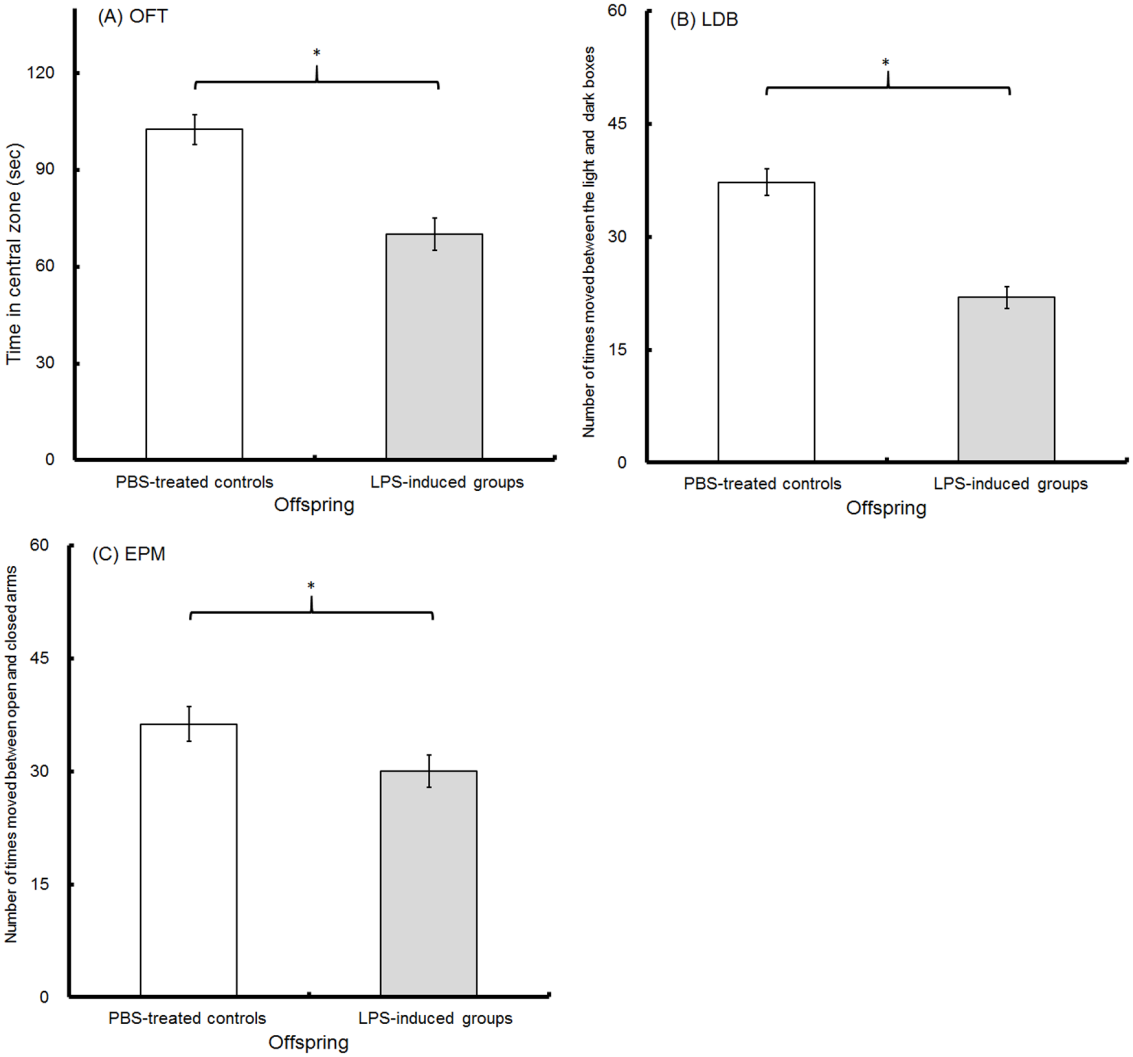
Anxiety-like behaviors. At 5 weeks old, the time (sec) in the central area of the open-field test (A, OFT, n = 30), the number of times that the mice moved between the light and dark boxes during the light/dark box (LDB) test at 6 weeks old (B, n = 30) and the number of times that the mice moved between the open and closed arms during the EPM test at 9 weeks old (C, n = 30) were recorded in the offspring. Between PBS-induced (hollow) and LPS-induced (solid) offspring, the significant differences (*) at p<0.05 are indicated (n = 30; t-test; two-tailed).

In the OFT, the 5-week-old LPS-induced offspring spent less time in the central zones (70.11±4.94 sec) than the PBS-treated offspring (102.49±4.58 sec) (Fig 3A; t-test, p<0.001). At 6 weeks of age, the offspring were evaluated with the LDB test. The LPS-induced offspring appeared fewer times (21.93±1.48) moving between the light and dark boxes than the PBS-treated offspring (37.27±1.73) (Fig 3B; t-test, p<0.001). At 9 weeks old, the offspring were subjected to the EPM tests. The number of times that the mice moved between the open and closed arms was 30.03±2.13 for the LPS-induced offspring and 36.30±2.29 for the PBS-treated offspring (Fig 3C, t-test, p = 0.049). Our OFT, LDB and EPM results indicate that anxiety-like behaviors were induced in LPS-induced offspring by prenatal maternal immune activation.

### mRNA expression

**Fig 4:**
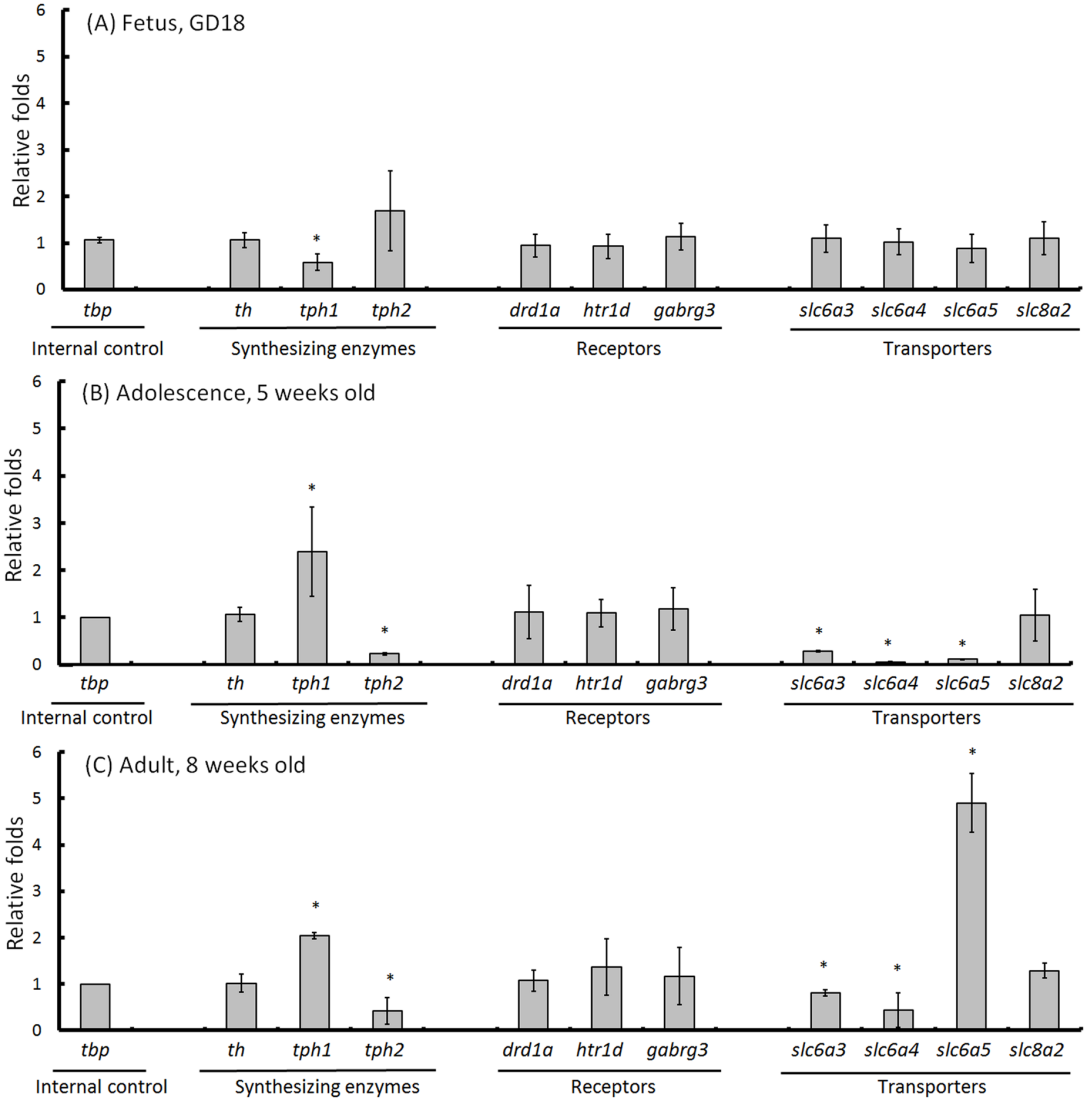
mRNA expression. The brain tissues were dissected from fetuses at GD18 (A, n = 3), adolescent mice at 5 weeks old (B, n = 3) and adult mice at 8 weeks old (C, n = 3). The specific mRNA expression in the cerebrum was detected by real-time PCR. The tbp (TATA sequence binding protein) gene was used as a reference gene and an internal control because tbp expression is stable in the mouse brain across different stages of development and different phases of LPS stimulation [24, 25]. Each gene (independent samples, n = 3) was first normalized to the tbp reference gene using the following equation: ΔCq = Cq target gene−Cq_*tbp* reference gene_. The changes in gene expression between PBS-treated controls and LPS-induced groups were calculated as follows: ΔΔCq = (Cq target gene in LPS-induced group−Cq_tbp reference gene in LPS-induced group_)–(Cq_target gene in PBS-treated control_−Cq_tbp reference gene in PBS-treated control_), and the relative fold = 0.5^ΔΔCq^. The significant differences (*) at p<0.05 are indicated (one-way ANOVA; Tukey’s post hoc test).

To determine the expression of neurotransmitter-related genes in the PBS-treated controls and LPS-induced offspring, the synthesizing enzyme (th for DA; tph1 and thp2 for 5-HT), receptor (drd1a for DA; htr1d for 5-HT; gabrg3 for γ-aminobutyric acid) and transporter (slc6a3 for DA; slc6a4 for 5-HT; slc6a5 for glycine; slc8a2 for sodium/calcium exchanger) genes were detected in brain tissue from fetuses at GD18 as well as in adolescent (5 weeks old) and adult (8 weeks old) mice. In the brain, the tph1 gene was predominately distributed in the pineal gland, whereas the tph2 gene was predominately distributed in the raphe nuclei [30]. With the exception of the tph1 gene, all tested genes in the fetal cerebrum were unchanged in both groups (Fig 4A). However, changes were detected in the synthesizing enzyme genes for 5-HT (tph1 and thp2) and transporters (slc6a3, slc6a4, and slc6a5) in the adolescent (Fig 4B) and adult (Fig 4C) offspring. Compared with the PBS-treated controls, the tph1 gene was increased and the tph2 gene was decreased in the adolescent and adult offspring; both DA (slc6a3) and 5-HT (slc6a4) transporter genes were decreased in adolescent and adult offspring, whereas the glycine transporter gene (slc6a5) was decreased in the adolescent but increased in the LPS-induced adult. No changes were detected in the expression levels of the neurotransmitter receptors (drd1a, htr1d and gabrg3) or the sodium/calcium exchanger gene (slc8a2) in the fetal, adolescent and adult brains between the PBS-treated controls and LPS-induced groups. Our results revealed that the expression levels of the 5-HT-related synthesizing enzyme (tph2) and transporter (slc6a4) genes were significantly depleted in parallel to the occurrence of anxiety-like behaviors in LPS-induced offspring.

### Morphology of neurons and glial cells

**Fig 5:**
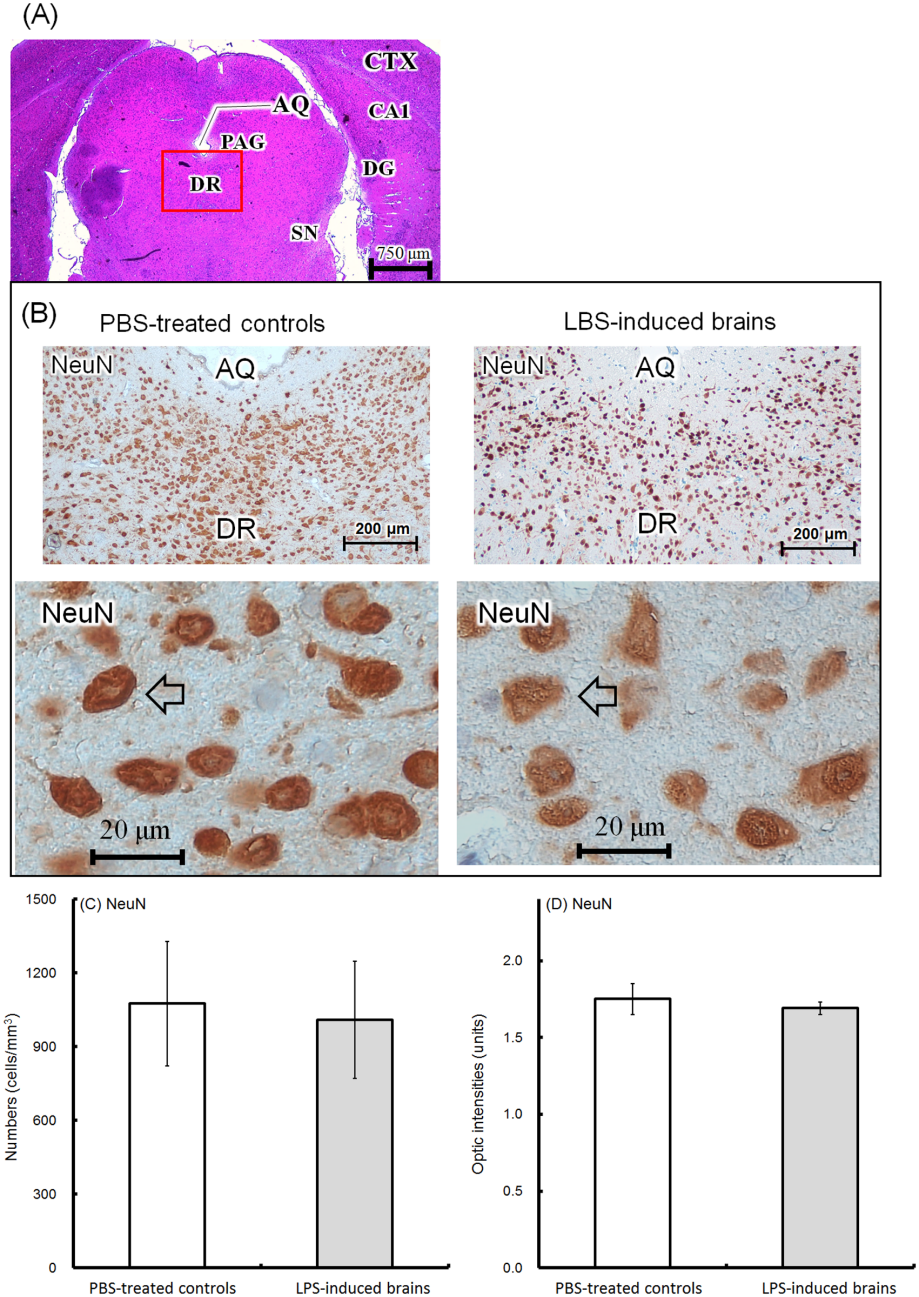
Anatomical position and histological examination of NeuN neurons. As the representative tissue, brains were collected from the 8-week-old offspring. The anatomical position of the cerebral cortex (CTX), CA1 region of the hippocampus (CA1), dentate gyrus of the hippocampus (DG), cerebral aqueduct (AQ), periaqueductal gray (PAG), dorsal raphe nuclei (DR) of the midbrain, and substantia nigra (SN) are shown (A, H&E stain, 20X). In the DR region, the NeuN-expressing neurons are shown (B, IHC; upper left, PBS-treated controls, 100X; upper right, LPS-induced brains, 100X; lower left, PBS-treated controls, 400X; lower right; LPS-induced brains, 400X; NeuN neuron, arrow indicated). The number of NeuN-positive neurons was determined using 10 random microscopic fields (200X) for each brain tissue. The average numbers (cells/mm^3^) in the PBS-treated controls (hollow; n = 6, independent tissues) and LPS-induced brains (solid; n = 6, independent tissues) are shown (C). The optical densities (units) of NeuN-positive areas were calculated using ImageJ software (open sources, https://imagej.net/Fiji). The average values in the PBS-treated controls (hollow; n = 6, independent tissues) and LPS-induced offspring (n = 6, independent tissues) are shown (D).

**Fig 6:**
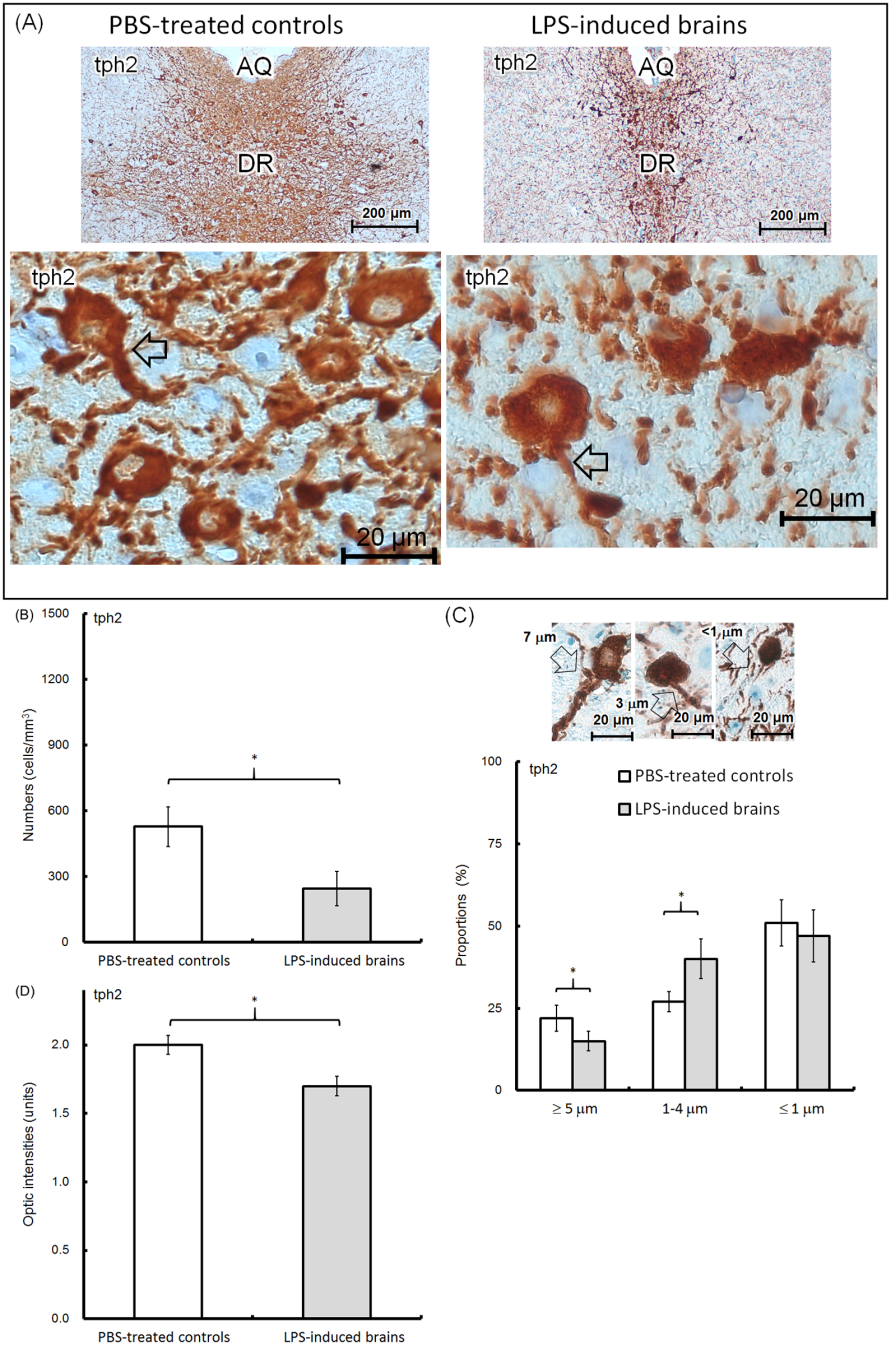
Histological examination of tph2-expressing neurons. Brain tissues were collected from the 8-week-old offspring. Using immunohistochemistry, the tph2-expressing neurons on the dorsal raphe nuclei (DR) are shown (A, upper left; PBS-treated controls;100X; upper right; LPS-induced brains; 100X; lower left; PBS-treated controls; 400X; lower right; LPS-induced brains; 400X; axon hillock, arrow indicated). The number of tph2-expressing neurons was determined using 10 random microscopic fields (200X) for each brain tissue. The average numbers (cells/mm^3^) in the PBS-treated controls (hollow; n = 6, independent tissues) and LPS-induced brains (solid; n = 6, independent tissues) are shown (B). Based on the diameter (≥5 μm, 2–4 μm and ≤1 μm) of the axon hillock, the three categories of neurons are shown (C, upper). The average percentage (%) of 5-HTergic axons in each category was determined using 100 neurons and shown (C, lower; n = 6, independent tissues; each category and group). The optic densities (units) of the tph2-positive area were calculated using ImageJ software (open sources, https://imagej.net/Fiji). The average values in the PBS-treated controls (hollow; n = 6, independent tissues) and LPS-induced brains (solid; n = 6, independent tissues) are shown (D). The significant differences (*) at p<0.05 are indicated (t-test).

**Fig 7:**
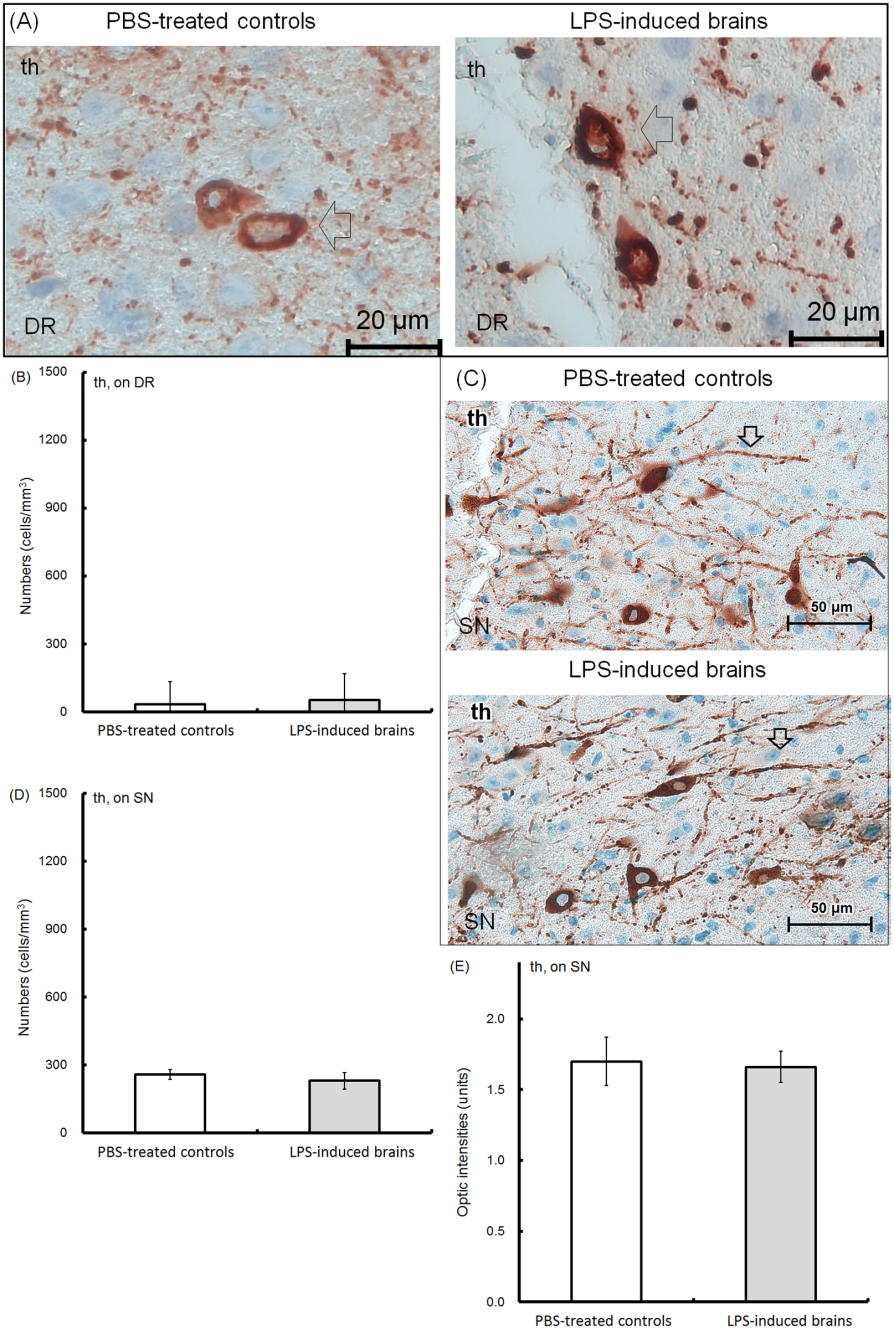
Histological examination of th-expressing neurons. Brain tissues were collected from the 8-week-old offspring. Using immunohistochemistry, representative th-expressing neurons in the dorsal raphe nuclei (DR) (A, left; PBS-treated controls; right; LPS-induced brains; 400X, arrow indicated). The number of th-expressing neurons on DR was determined using 10 random microscopic fields (200X) for each brain tissue. The average numbers (cells/mm^3^) of th-positive cells in the PBS-treated controls (hollow; n = 6, independent tissues) and LPS-induced brains (solid; n = 6, independent tissues) are shown (B). On substantia nigra (SN), the th-expressing neurons and fibers are shown (C, upper, PBS-treated controls; lower, LPS-induced brains; 400X; th-expressing fibers, arrow indicated). The number of th-expressing neurons on SN was determined using 10 random microscopic fields (200X) for each brain tissue. The average numbers (cells/mm^3^) of th-expressing neurons in the PBS-treated controls (hollow; n = 6, independent tissues) and LPS-induced brains (solid; n = 6, independent tissues) are shown (D). The optic densities (units) of the th-expressing areas on SN were calculated using ImageJ software (open sources, https://imagej.net/Fiji). The average values in the PBS-treated controls (hollow; n = 6, independent tissues) and LPS-induced brains (solid; n = 6, independent tissues) are shown (E).

Brain tissues were collected from the offspring at 8 weeks of age. We obtained anatomically identical positions of the cerebral cortex (CTX), hippocampus (CA1 region and dentate gyrus [DG]), cerebral aqueduct (AQ), periaqueductal gray (PAG), dorsal raphe nuclei (DR) of the midbrain, and substantia nigra (SN) from each slide (Fig 5A). In the DR, the total number of NeuN-expressing neurons is shown in Fig 5B (PBS-treated controls, upper left, 100X; lower left, 400X; LPS-induced offspring, upper right, 100X; lower right, 400X). The number of NeuN-expressing neurons on DR was approximately 1075±252 cells/mm^3^ in the PBS-treated controls, but 1009±239 cells/mm^3^ in the LPS-induced brains (Fig 5C). Optic density (OD) of the area containing the NeuN-immunoreactive cells in the PBS-treated controls was 1.75±0.1 units, which was similar to that of the LPS-induced brains (1.69±0.04 units; Fig 5D). The numbers or distributed intensities of NeuN neurons were identical between the controls and LPS-induced groups. IHC revealed that tph2-expressing neurons were predominately localized in DR (Fig 6A, PBS-treated controls, upper left, 100X; lower left, 400X; LPS-induced offspring, upper right, 100X; lower right, 400X). However, there were 528±90 cells/mm^3^ of 5-HTergic neurons in the DR of PBS-treated controls, which was higher than that in the LPS-induced brains (246±79 cells/mm^3^) (Fig 6B). The 5-HTergic neuron axons (>5 μm) in the LPS-induced brains were relatively thinner than those (<5 μm) in the PBS-treated controls (Fig 6A, lower left, PBS-treated controls; lower right, LPS-induced brains; arrow indicated). The diameter of the axon hillock in the 5-HTergic neurons was categorized into three groups (≥5 μm, 2–4 μm, ≤1 μm). There were 22±4%, 27±3% and 51±7% in the ≥5 μm, 2–4 μm and ≤1 μm groups, respectively, in the PBS-treated control brains, whereas there were 15±3%, 40±6% and 47±8%, respectively, in the LPS-induced brains (Fig 6C). The OD of the tph-2-positive area in the PBS-treated controls was 2.0±0.07 units, which was higher than the OD of the positive area in the LPS-induced brains (1.7±0.07 units) (Fig 6D). According to our results, the cell numbers, distributed densities and the sizes of the axon hillock of the 5-HTergic neurons were significantly depleted in LPS-induced brains. The number of th-expressing cells and fibers in the DR (left, PBS-treated controls; right, LPS-induced brains) are shown in Fig 7A. Because DAergic neurons are rarely distributed in the DR, it was not surprising that the th-expressing cells were 34±22 cells/mm^3^ in PBS-treated controls and 55±37 cells/mm^3^ in LPS-induced brains (Fig 7B). More th-expressing cells and fibers were found in SN (Fig 7C, upper, PBS-treated controls; lower, LPS-induced brains; DAergic fibers, arrow indicated). There were 259±100 cells/mm^3^ in PBS-treated controls and 231±116 cells/mm^3^ in LPS-induced groups (Fig 7D). Approximately 1.7±0.17 and 1.66±0.11 units of OD were found in the SN of the PBS-treated and LPS-induced brains, respectively (Fig 7E). However, there were not differences in the cell numbers and/or ODs in the DR and SN between the PBS-treated controls and LPS-induced groups.

**Fig 8:**
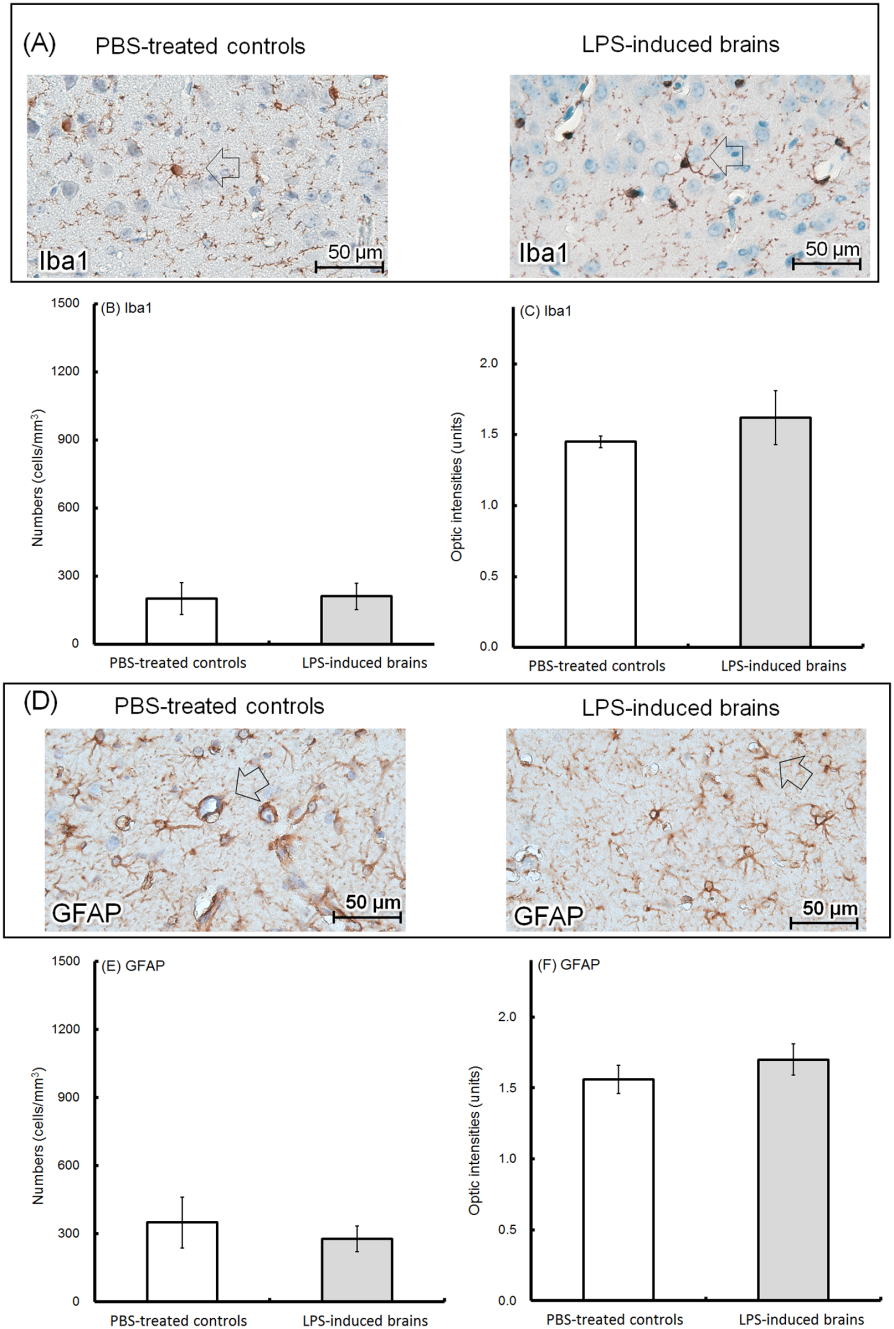
Histological examination of glial cells. Brains were collected from the 8-week-old offspring. Using immunohistochemistry, representative Iba1-positive microglia in the cerebral cortex are shown (A, left; PBS-treated controls; right; LPS-induced brains; arrow indicated; 400X). The number of Iba1-positive microglia was determined using 10 random microscopic fields (200X) for each brain tissue. The average numbers (cells/mm^3^) of Iba1-positive microglia in the PBS-treated controls (hollow; n = 6, independent tissues) and LPS-induced brains (solid; n = 6, independent tissues) are shown (B). The optic density (units) of the Iba1-expressing areas was calculated using ImageJ software (open sources, https://imagej.net/Fiji). The average values in the PBS-treated controls (hollow; n = 6, independent tissues) and LPS-induced brains (solid; n = 6, independent tissues) are shown (C). GFAP-positive astrocytes in the surrounding perivascular cuffs of the hippocampus are shown (D, left; PBS-treated controls; right; LPS-induced brains; arrow indicated; 400X). The number of GFAP-expressing astrocytes was determined using 10 random microscopic fields (200X) for each brain tissue. The average numbers (cells/mm^3^) of GFAP-expressing astrocytes in the PBS-treated controls (hollow; n = 6, independent tissues) and LPS-induced brains (solid; n = 6, independent tissues) are shown (E). The optic densities (units) of the GFAP-expressing areas were calculated using ImageJ software. The average values in the PBS-treated controls (hollow; n = 6, independent tissues) and LPS-induced brains (solid; n = 6, independent tissues) are shown (F).

In this study, typical Iba1-positive microglia with round to oval cell bodies and thin ramified branches were observed on CTX (Fig 8A, left; PBS-treated controls; right; LPS-induced brains; microglia, arrow indicated). Hypertrophied or swelling of activated microglia were rarely seen in the two groups. The number of microglia was 203±71 cells/mm^3^ (Fig 8B), and the OD of the Iba1-positive areas was1.45±0.04 units (Fig 8C) in the PBS-treated controls, whereas in the LPS-induced brains, the number of microglia was 213±58 cells/mm^3^ (Fig 8B), and the OD of the Iba1-positive areas was 1.62±0.19 units (Fig 8C). Typical GFAP-positive astrocytes in the surrounding perivascular cuffs appeared in both groups (Fig 8D, left, PBS-treated controls; right, LPS-induced brains; astrocytes, arrow indicated). In the hippocampus, the number of astrocytes was 350±112 cells/mm^3^ in the PBS-treated controls and 278±57 cells/mm^3^ in the LPS-induced brains (Fig 8E) and the ODs of the GFAP-positive areas was 1.56±0.1 units in the PBS-treated controls and 1.7±0.11 units in the LPS-induced groups (Fig 8F). Hypertrophied cell bodies and thicker processes were not observed in the GFAP-positive astrocytes in either group. In addition, astrogliosis was not observed. No difference in morphology, cell numbers and immunoreactive densities was observed in the glial cells of both groups.

### Expression of 5-HT and DA in the cerebrum

**Fig 9:**
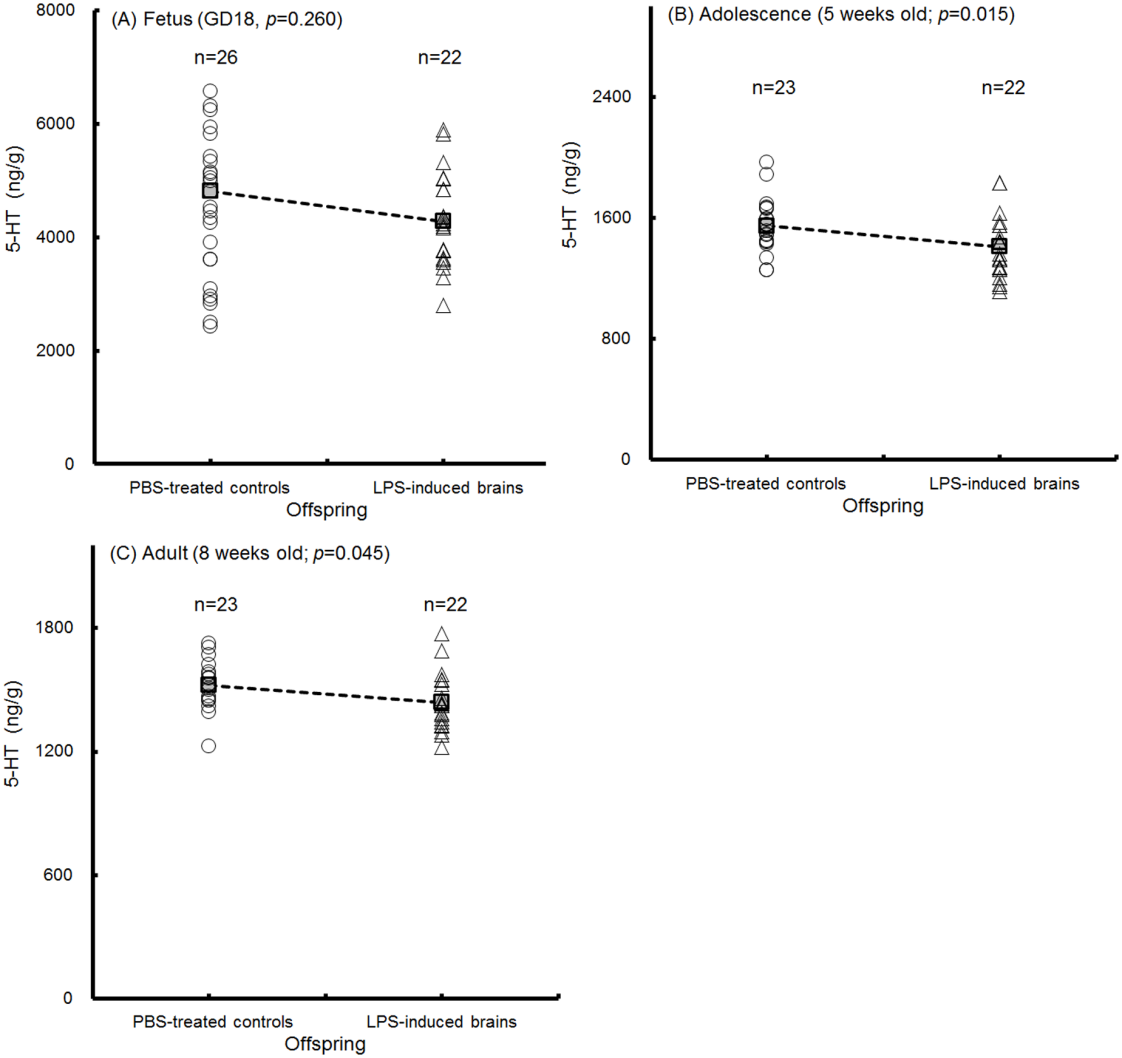
Expression of cerebral serotonin. The serotonin (5-HT) levels in homogenized brains from the offspring were detected. The cerebral 5-HT levels (ng/g) in fetuses at GD18 (A, n = 26, PBS-treated controls; n = 22, LPS-induced offspring), adolescent mice at 5 weeks old (B, n = 23, PBS-treated controls; n = 22, LPS-induced offspring) and adult mice at 8 weeks old (C, n = 23, PBS-treated controls; n = 22, LPS-induced offspring) are shown. The means are indicated by the dotted line. The p-value was determined by two-tailed t-test.

**Fig 10:**
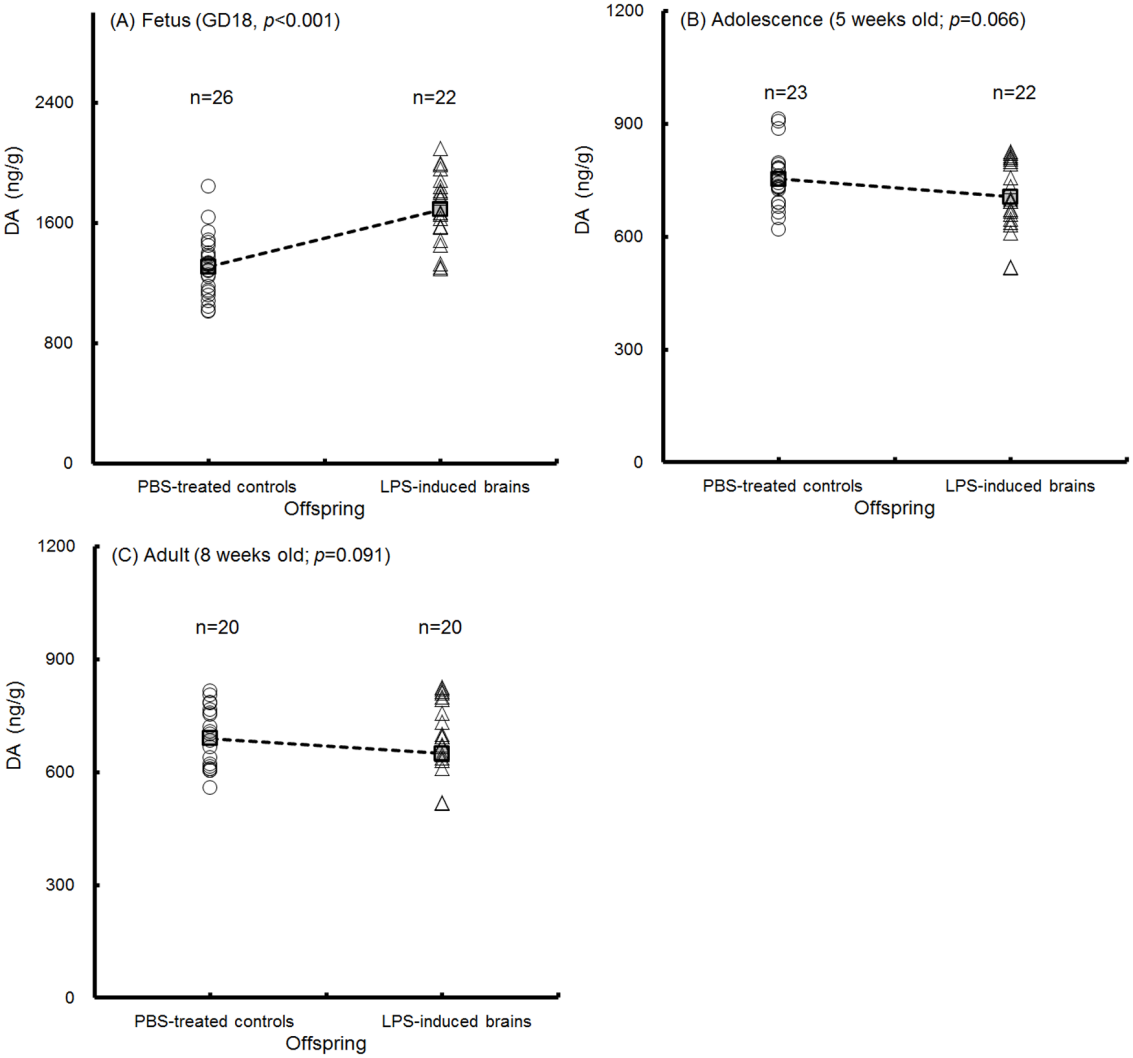
Expression of cerebral dopamine. The dopamine (DA) levels in homogenized brains from the offspring were detected. The cerebral DA levels (ng/g) in fetuses at GD18 (A, n = 26, PBS-treated controls; n = 22, LPS-induced offspring), adolescent mice at 5 weeks old (B, n = 23, PBS-treated controls; n = 22, LPS-induced offspring) and adult mice at 8 weeks old (C, n = 20, PBS-treated controls; n = 20, LPS-induced offspring) are shown. The means are indicated by the dotted line. The p value was determined by two-tailed t-test.

All of the homogenized brain tissues contained the CTX, hippocampus, midbrain (DR and SN), hind brain and brain stem. The concentration of cerebral 5-HT and DA in the offspring was detected. No difference in 5-HT concentrations of fetal brains at GD18 was detected between the PBS-treated controls and LPS-induced offspring (Fig 9A; p = 0.260); however, 5-HT levels were significantly decreased in the adolescent (Fig 9B; p = 0.015) and adult (Fig 9C, p = 0.045) brains of the mice in the LPS-induced groups. Cerebral DA was significantly elevated in fetal brains at GD18 in the LPS-induced offspring (Fig 10A; p<0.001). In the adolescent (Fig 10B; p = 0.066) and adult (Fig 10C; p = 0.091) offspring, cerebral DA trended toward a decrease in the LPS-induced group compared with that in the PBS-treated control. Our results indicated that the concentration of the cerebral 5-HT and gene expression of the tph2 and slc6a4 were decreased in conjunction with the appearance of anxiety-like behaviors in the adolescent and adult offspring of the LPS-induced groups.

## Discussion

**Fig 11:**
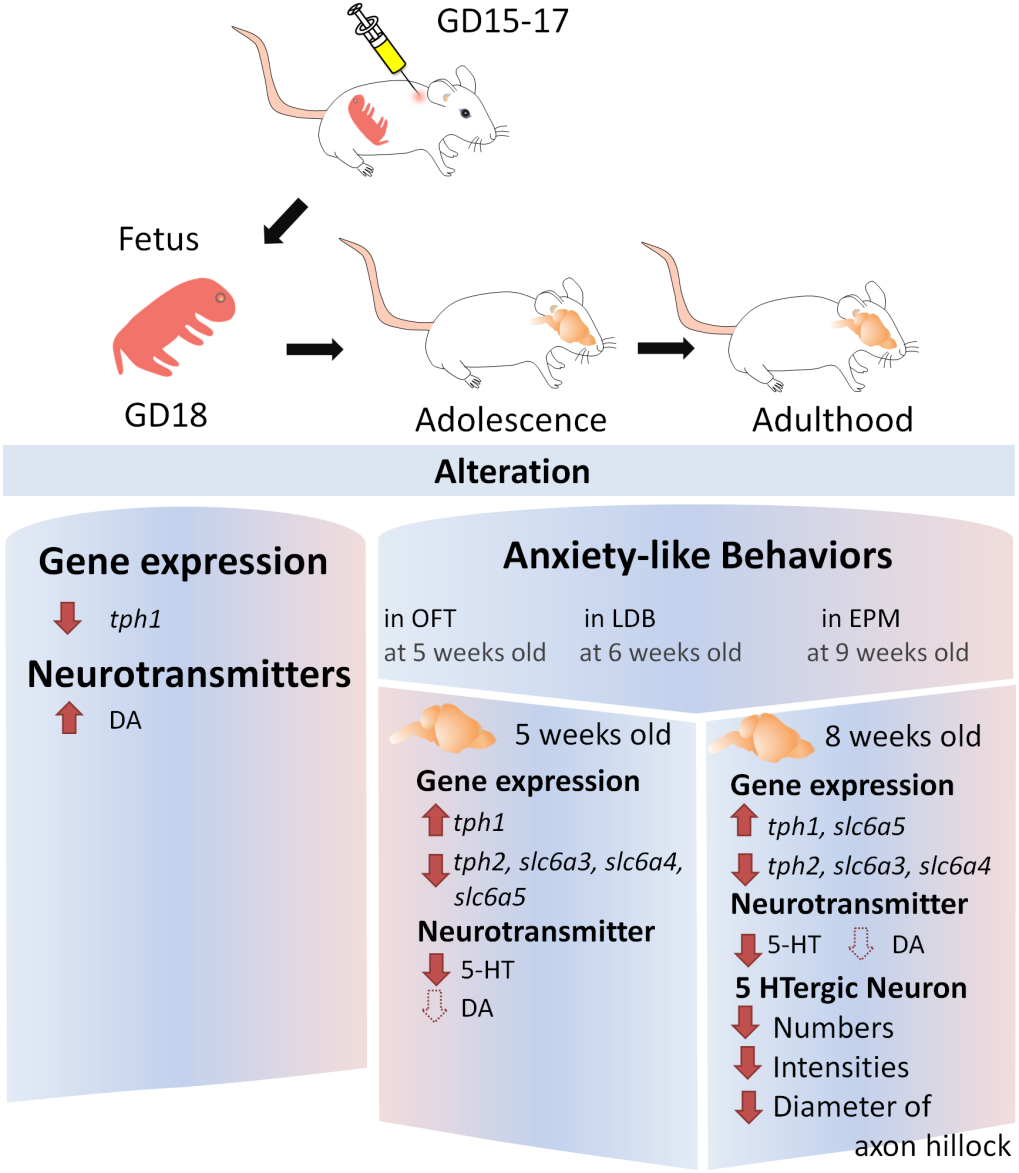
Summary of the effect of maternal immune activation on serotonin and dopamine. Pregnant mice were subcutaneously injected with lipopolysaccharide (LPS) at GD15 (25 μg/Kg), GD16 (25 μg/Kg) and GD17 (50 μg/Kg). Compared to the PBS-treated controls, the mRNA level of the tph1 gene (an isoform of serotonin synthesizing enzyme expressed on pineal gland) was down-regulated and the DA levels were elevated in the LPS-induced female fetal brains at GD18. In the female offspring, anxiety-like behaviors were observed in the OFT at 5 weeks old, in the LDB test at 6 weeks old, and in the EPM at 9 weeks old. At 5 weeks old, the mRNA level of the tph1 gene was up-regulated, whereas that of the tph2 gene (another serotonin synthesizing enzyme isoform mainly expressed in the raphe nuclei of the midbrain), slc6a3 (a DA transporter), slc6a4 (a 5-HT transporter) and slc6a5 (a glycine transporter) as well as the 5-HT levels were down-regulated in the cerebrum of the female offspring. At 8 weeks old, the tph1 and slc6a5 genes were up-regulated, and the tph2, slc6a3 and slc6a5 genes were down-regulated in the brains of the female offspring. The cerebral 5-HT levels were significantly decreased at 5 and 8 weeks old (solid arrow), whereas the DA levels exhibited a trend towards a decrease (dotted arrow). Moreover, the total number and distribution of tph2-expressing 5-HTergic neurons, and the diameter of the 5-HTergic axon hillocks were decreased at the age of 8 weeks. We concluded that maternal immune activation induced by exposure to a low dose of LPS decreased cerebral 5-HT in parallel with the down-regulation of 5-HT-related genes (tph2 and slc6a4), resulting in anxiety-like behavior in the female offspring.

As summarized in Fig 11, pregnant C57BL/6 mice exposed to LPS from GD15 to GD17 can induce anxiety-like behaviors in female offspring. After maternal immune activation, down-regulation of the tph1 gene (an isoform of the 5-HT synthesizing enzyme expressed in the pineal gland) and up-regulation of DA levels appeared in the fetal brain at GD18. In terms of long-term effects, the tph2 (an isoform of the 5-HT synthesizing enzyme mainly expressed in the DR of midbrain), slc6a3 (a DA transporter) and slc6a4 (a 5-HT transporter) mRNA levels as well as the 5-HT levels were down-regulated in the adolescent (5 weeks old) and adult (8 weeks old) brains (Fig 11).

The tph1 gene in the CNS is mainly expressed in the pineal gland and controls the rate of 5-HT production by serving as a precursor of melatonin, which mediates the circadian rhythm of the sleep-wake cycle [30–32]. Down-regulation of the tph1 gene in the fetal brain indicates an imbalance of the melatonin system, which may be involved in the sleep disturbances and circadian rhythm disorganization that usually appear in patients with psychiatric conditions such as depression, autism and Alzheimer’s disease [1, 33, 34]. The tph2 gene is expressed on 5-HTergic neurons in the raphe nuclei of the midbrain [35]. The tph2 neurons of 5-HT system project to the hippocampus, septum, hypothalamus, striatum and cortex and plays roles in sleep, arousal, learning and memory, sensory perception, motor coordination, pain regulation and ingestive behaviors [36]. Typically 5-HTergic axons on DR are large (up to 5 μm in diameter) and make synaptic connections with postsynaptic neurons [34]; however, in this study, due to the long-term effects of maternal immune activation, thinner 5-HTergic axons (<5 μm) were found in the brain of the 8-week-old offspring, indicating that the activity of 5-HTergic neurons was decreased in LPS-induced offspring. A number of markers such as 5-HT synthesizing enzymes, transporters and receptors that are involved in 5-HTergic activities have been suggested to link anxiety and autism in human and animals [3–7]. The 5-HT levels are controlled via the serotonin transporter (slc6a4), which is responsible for serotonin reuptake from the synaptic cleft to the presynaptic neuron [8, 37]. The facts including appearance of thinner 5-HTergic axons and down-regulation of the 5-HT synthesizing enzyme (tph2) and transporter (slc6a4) genes agreed that resultant 5-HT levels in LPS-induced brains were decreased (Fig 11). Tph1 in the periphery produces higher levels of 5-HT than in neurons, but in the CNS, tph2-expressing cells are more abundant than tph1 cells [38, 39]. Thus, in this study, the tph1 gene was up-regulated in the LPS-induced groups but cannot compensate for the reduced total 5-HT levels (Fig 11).

LPS is an endotoxin, a strong immunomodulator that raises maternal inflammatory cytokines (TNF-α, IL-1 and IL-6) and corticosterone (an indicator of HPA activation in response to LPS-induced stress) [40, 41]. However, LPS is not a placenta-transmissible molecule [40]. The effect of maternal immune activation on fetal brains relies on the interplay among maternal transmissible cytokine IL-6 and cortisol as well as fetal effector immune cells, glial cells and neurons [40, 42]. The resultant interactions directly or indirectly increase 5-HTergic and DAergic activity, which peaked at 6 h and persisted until 24 h [17, 40, 43]. In our model, the short-term effect (24 h) of maternal immune activation significantly induced augmentation of DA levels in the fetal brains (Fig 11). Cognate DA receptors as well as macrophages, naïve and activated T cells and resting NK cells have been abundant in the striatum and frontal cortex during fetal brain development [44, 45]. DA contributes to DAergic neuronal projections and also mediates the release of inflammatory cytokines (IL-2, IFN-γ and IL-6) that have been shown to regulate neuronal development through the activation of macrophages/microglia [44]. The telencephalon, diencephalon, midbrain, hindbrain and spinal cord are developed at GD15 (it is commencement of the LPS injections in this study). Furthermore, the granular cells within hippocampal dentate gyrus, the last structure formed during hippocampal development, appear at postnatal day 10 [46]. In this study, the elevated DA levels in the fetal brains indicates the occurrence of aberrant cytokine expression and neuronal development, which ultimately results in an imbalance of 5-HT synthesizing enzymes (tph1 and tph2) and DA (slc6a3) or 5-HT (slc6a4) transporters in the adult brains through glial-neuron, cytokine-neuron and transmitter-neuron interactions (Fig 11).

Both γ-aminobutyric acid (GABA) and glycine are the major inhibitory neurotransmitters in the CNS. The glycine transporter (slc6a5) plays a role in sustaining and prolonging glycine transmission through presynaptic reuptake and vesicle reloading in inhibitory presynaptic terminals [47]. No report to date has indicated an association between slc6a5 gene expression and the appearance of anxiety-like behavior; however, mice with slc6a5 homozygous or heterozygous defects exhibit grooming and hyperactive behaviors that are typically observed in mouse models of schizophrenia [48]. In this study, we found that the expression of slc6a5 (glycine transporter) fluctuated in the adolescent (down-regulation) and adult (up-regulation) brains (Fig 11). The GABA receptor gabrg3 is localized at chromosome 15q11-q13, a region that has been reported to have significant linkage disequilibrium with an ASD susceptibility locus [49]. The slc8a2 gene, a Na/Ca^2+^ exchanger, regulates the transient elevation of intracellular calcium signaling induced by robust glial and neuronal activation [50]. The deletion of chromosome 19q13.32, which harbors the slc8a2 gene, results in the occurrence of intellectual disabilities, facial asymmetry and oculomotor paralysis [51]. However, in this study, no alterations in gabrg3 and slc8a2 gene expression were found. We agreed that neither gabrg3 nor slc8a2 single locus did not directly affect the anxiety- and ASD-like behaviors [50, 51].

Astrocytes can regulate neuronal synapse formation, myelination and homeostasis in the CNS. After LPS exposure, the protein levels and immunoreactivity of GFAP (an astrocyte marker) but not Iba1 (a microglial marker) were reported to be increased in neonate brains [52], indicating that glial-neuronal interactions could be altered. However, as a long-term effect in the adulthood offspring, the cell numbers, distributed intensities or the morphology of GFAP-positive astrocytes and of Iba1-positive microglia were identical to controls. Microglia had round to oval cell bodies and ramified branches. Astrocytes were typically localized in the surrounding perivascular cuffs. Activated microglia and astrogliosis have been reported in severe neurodegenerative disorders such as schizophrenia, Alzheimer’s disease or Parkinson’s disease [53, 54] but were not observed in our maternal immune activation model.

Unpredictable stress occurring during adolescence can alter the concentration of cortical 5-HT and anxiety-related behavior in adulthood but has no effect on DA levels in the brain [55]. We conducted behavioral tests that likely exposed the mice to unpredictable stress every week. Thus, the unpredictable stress that occurred during the behavioral tests could induce an imbalance in cerebral neurotransmitters. However, compared to the PBS-treated offspring, 5-HT and its related genes were decreased in the LPS-induced adolescent and adult offspring. The cerebral DA levels showed a decreasing trend although the mRNA levels of th (DA synthesizing enzyme) and dard1a (DA receptor) genes were not altered from adolescence to adulthood. Aberrant cerebral striatal and prefrontocortical DA and 5-HT expression associated with anxiety and hypoactivity for the mice repeatedly exposed to LPS [56]. Thus, 5-HT or DA deficiency in the brain might be an important causative factor of anxiety- and ASD-like behaviors in humans or animal models [6, 9, 57, 58].

The exposure of pregnant mice to high doses of LPS (≥10 mg/Kg) causes a robust inflammatory response in the brain *via* toll-like receptor 4 signaling that initially occurs in circumventricular organs and choroid plexus [59]. However, severe endotoxemia frequently leads to abortion in pregnant mice [60]. We found that an injection of >100 μg/Kg LPS resulted in an abortion in approximately 60–66% of the pregnant mice. Low dose (≤100 μg/Kg) of LPS induces maternal immune activation similar to that of a chronic infection or contact with environmental allergens such as dust [16, 19, 61]. Using low-dose LPS stimulation, the rate of abortion was as low as 11% in this study. Infections or the exposure to allergens in pregnant women have been linked to anxiety and ASD in their adult offspring [62, 63]. Moreover, in terms of the clinical perspectives of human subjects, anxiety and depression occur more often in females, but ASD-like behaviors occur more frequently in males [55, 64]. Thus, the female offspring of the maternal immune activation model, which was induced by repeated injections of low dose LPS (25–50 μg/Kg, per injection; sc), were used to observe the incidence of anxiety-like behaviors in this study.

Despite the novel insights into neurodevelopmental and behavioral changes in offspring provided by the maternal immune activation model, the results of some experiments have not been reproduced in studies between different laboratories [19, 21, 63]. For example, Babri et al. have reported that maternal immune activation induced by exposure to LPS (ip, 500 μg/Kg; at GD17) resulted in no changes in stress-induced corticosterone levels and no differences in anxiety-like behaviors in male offspring [21]. However, in Depino’s experiment, male offspring exhibited anxiety-like behaviors after maternal immune activation (ip, 25 μg/Kg LPS; at GD9) [23]. This difference is likely due to differences in the exposure time, dose of LPS and, importantly, the experimental conditions. Under Depino’s conditions (maternal immunization at GD9; tests for male offspring), anxiety-like behaviors were also observed. Nevertheless, changes in locomotive ability (OFT, at adolescence) and muscular strength (WH test, at adult) did not occur in our study. Depino et al. reported that the locomotive ability in the OFT differed between the LPS- and PBS-treated groups, but they did not estimate muscle strength [23].

However, developing a C57BL/6 mouse model using prenatal exposure to LPS allowed us to longitudinally examine the development of anxiety-like behavioral changes and determine the marker of autism in prenatal to adult mice.

## Supporting information

S1 Fig Cross-reactivity and linearity of the 5-HT ELISA vs HPLC and DA ELISA vs HPLC. The brain tissues (containing the intact cerebral cortex, hippocampus, substantia nigra, midbrain, hind brain and brain stem) were weighed, homogenized and deproteinized in 300 μL of a 0.2 N perchloric acid solution. After centrifugation (14,000 x g for 30 min at 4°C), the supernatants were filtered through 0.2 μm membrane and analyzed using high pressure liquid chromatography (5 μL; HPLC, Hitachi Chromaster System, Petrzikova, Praha, Czech Republic) equipped with a reverse-phase column (Thermo Scientific Syncronis C18, 5.0 μm, 4.6 x 250 mm; Fisher Scientific Co., PA, USA), colorimetric detector (L-2400, Hitachi Co.), and 6011RS ultra analytical cell and pump (L-2130, Hitachi Co.). The mobile phase was 75 mM Na_2_HPO_4_, 1.7 mM 1-octanesulfonic acid, 100 μL/L triethylamine, 25 μM ethylenediaminetetraacetic acid and 10% (v/v) acetonitrile (pH 3.0), and the flow rate was 0.5 mL/min. The concentration of both 5-HT and DA was determined using the areas of the sample peaks against the areas of the reference 5-HT and DA standards (Sigma Co.) at a specific retention time (5-HT at 6.38 min; DA at 13.28 min). For the ELISA, the brain tissues were prepared in 300 μL of PBS (see Materials and methods). The OD values of 5-HT, 5-hydroxyindoleacetic acid (5-HIAA), melatonin and tryptamine, at concentrations ranging from 15 ng/mL to 240 ng/mL, as measured by ELISA at 450 nm, are shown (A). The OD values of DA, adrenaline, noradrenaline, L-dopa, tyramine and homovanillic acid, at concentrations ranging from 15 ng/mL to 120 ng/mL, as measured by ELISA at 450 nm, are shown (B). A total of 10 representative brain tissues (n = 5, LPS-induced brains; n = 5, PBS-treated controls) were homogenized with 300 μL PBS and separated into two equal volumes (150 μL for each). One was diluted into 300 μL PBS for ELISA and another was diluted into a final concentration of 0.2 N perchloric acid solutions (300 μL) for HPLC. The concentration (ng/g) of the neurotransmitter each paired sample was respectively determined by ELISA and HPLC. The linearity of 5-HT (C) and DA (D) detected by both methods are shown.
(TIF)

S1 Table Dose effects of LPS on the abortion and survival of offspring after maternal immune activation. (DOCX)

## References

[1] Matson JL, Rieske RD, Williams LW. The relationship between autism spectrum disorders and attention-deficit/hyperactivity disorder: an overview. Res Dev Disabil. 2013;34(9):2475–84. Epub 2013/06/12. doi: 10.1016/j.ridd.2013.05.021 . 23751293 10.1016/j.ridd.2013.05.021

[2] Kerns CM, Kendall PC, Berry L, Souders MC, Franklin ME, Schultz RT, et al Traditional and atypical presentations of anxiety in youth with autism spectrum disorder. Journal of autism and developmental disorders. 2014;44(11):2851–61. Epub 2014/06/07. doi: 10.1007/s10803-014-2141-7 . 24902932 10.1007/s10803-014-2141-7 PMC5441227

[3] Ornoy A, Weinstein-Fudim L, Ergaz Z. Genetic Syndromes, Maternal Diseases and Antenatal Factors Associated with Autism Spectrum Disorders (ASD). Front Neurosci. 2016;10:316 Epub 2016/07/28. doi: 10.3389/fnins.2016.00316 ; 27458336 10.3389/fnins.2016.00316 PMC4933715

[4] Beaulieu JM, Zhang X, Rodriguiz RM, Sotnikova TD, Cools MJ, Wetsel WC, et al Role of GSK3 beta in behavioral abnormalities induced by serotonin deficiency. Proceedings of the National Academy of Sciences of the United States of America. 2008;105(4):1333–8. Epub 2008/01/24. doi: 10.1073/pnas.0711496105 ; 18212115 10.1073/pnas.0711496105 PMC2234138

[5] Coutinho AM, Sousa I, Martins M, Correia C, Morgadinho T, Bento C, et al Evidence for epistasis between SLC6A4 and ITGB3 in autism etiology and in the determination of platelet serotonin levels. Human genetics. 2007;121(2):243–56. Epub 2007/01/05. doi: 10.1007/s00439-006-0301-3 . 17203304 10.1007/s00439-006-0301-3

[6] Fernandez SP, Gaspar P. Investigating anxiety and depressive-like phenotypes in genetic mouse models of serotonin depletion. Neuropharmacology. 2012;62(1):144–54. Epub 2011/09/29. doi: 10.1016/j.neuropharm.2011.08.049 . 21945798 10.1016/j.neuropharm.2011.08.049

[7] Lowry CA, Johnson PL, Hay-Schmidt A, Mikkelsen J, Shekhar A. Modulation of anxiety circuits by serotonergic systems. Stress (Amsterdam, Netherlands). 2005;8(4):233–46. Epub 2006/01/21. doi: 10.1080/10253890500492787 . 16423712 10.1080/10253890500492787

[8] Voineagu I, Yoo HJ. Current progress and challenges in the search for autism biomarkers. Disease markers. 2013;35(1):55–65. Epub 2013/10/30. doi: 10.1155/2013/476276 ; 24167349 10.1155/2013/476276 PMC3774962

[9] Zarrindast MR, Khakpai F. The Modulatory Role of Dopamine in Anxiety-like Behavior. Archives of Iranian medicine. 2015;18(9):591–603. Epub 2015/09/01 . 26317601

[10] Eskow Jaunarajs KL, George JA, Bishop C. L-DOPA-induced dysregulation of extrastriatal dopamine and serotonin and affective symptoms in a bilateral rat model of Parkinson's disease. Neuroscience. 2012;218:243–56. Epub 2012/06/05. doi: 10.1016/j.neuroscience.2012.05.052 ; 22659568 10.1016/j.neuroscience.2012.05.052 PMC3393811

[11] Stansley BJ, Yamamoto BK. L-dopa-induced dopamine synthesis and oxidative stress in serotonergic cells. Neuropharmacology. 2013;67:243–51. Epub 2012/12/01. doi: 10.1016/j.neuropharm.2012.11.010 ; 23196068 10.1016/j.neuropharm.2012.11.010 PMC3638241

[12] Takeshima M, Miyazaki I, Murakami S, Kita T, Asanuma M. l-Theanine protects against excess dopamine-induced neurotoxicity in the presence of astrocytes. Journal of clinical biochemistry and nutrition. 2016;59(2):93–9. Epub 2016/10/05. doi: 10.3164/jcbn.16-15 ; 27698535 10.3164/jcbn.16-15 PMC5018574

[13] Yan QS, Yan SE. Activation of 5-HT(1B/1D) receptors in the mesolimbic dopamine system increases dopamine release from the nucleus accumbens: a microdialysis study. European journal of pharmacology. 2001;418(1–2):55–64. Epub 2001/05/04. . 11334865 10.1016/s0014-2999(01)00913-x

[14] Valentini V, Piras G, De Luca MA, Perra V, Bordi F, Borsini F, et al Evidence for a role of a dopamine/5-HT6 receptor interaction in cocaine reinforcement. Neuropharmacology. 2013;65:58–64. Epub 2012/09/18. doi: 10.1016/j.neuropharm.2012.08.025 . 22982249 10.1016/j.neuropharm.2012.08.025

[15] Gu F, Chauhan V, Chauhan A. Monoamine oxidase-A and B activities in the cerebellum and frontal cortex of children and young adults with autism. Journal of neuroscience research. 2017 Epub 2017/02/06. doi: 10.1002/jnr.24027 . 28151561 10.1002/jnr.24027

[16] Hsueh PT, Liu CL, Wang HH, Ni WF, Chen YL, Liu JK. A comparison of the immunological potency of Burkholderia lipopolysaccharides in endotoxemic BALB/c mice. Microbiology and immunology. 2016;60(11):725–39. Epub 2016/11/20. doi: 10.1111/1348-0421.12450 . 27862204 10.1111/1348-0421.12450

[17] Sens J, Schneider E, Mauch J, Schaffstein A, Mohamed S, Fasoli K, et al Lipopolysaccharide administration induces sex-dependent behavioural and serotonergic neurochemical signatures in mice. Pharmacology, biochemistry, and behavior. 2017;153:168–81. Epub 2017/01/07. doi: 10.1016/j.pbb.2016.12.016 . 28057525 10.1016/j.pbb.2016.12.016

[18] Karrow NA. Activation of the hypothalamic-pituitary-adrenal axis and autonomic nervous system during inflammation and altered programming of the neuroendocrine-immune axis during fetal and neonatal development: lessons learned from the model inflammagen, lipopolysaccharide. Brain, behavior, and immunity. 2006;20(2):144–58. Epub 2005/07/19. doi: 10.1016/j.bbi.2005.05.003 . 16023324 10.1016/j.bbi.2005.05.003

[19] Boksa P. Effects of prenatal infection on brain development and behavior: a review of findings from animal models. Brain, behavior, and immunity. 2010;24(6):881–97. Epub 2010/03/17. doi: 10.1016/j.bbi.2010.03.005 . 20230889 10.1016/j.bbi.2010.03.005

[20] Xuan IC, Hampson DR. Gender-dependent effects of maternal immune activation on the behavior of mouse offspring. PloS one. 2014;9(8):e104433 Epub 2014/08/12. doi: 10.1371/journal.pone.0104433 ; 25111339 10.1371/journal.pone.0104433 PMC4128679

[21] Babri S, Doosti MH, Salari AA. Strain-dependent effects of prenatal maternal immune activation on anxiety- and depression-like behaviors in offspring. Brain, behavior, and immunity. 2014;37:164–76. Epub 2013/12/12. doi: 10.1016/j.bbi.2013.12.003 . 24326014 10.1016/j.bbi.2013.12.003

[22] Hava G, Vered L, Yael M, Mordechai H, Mahoud H. Alterations in behavior in adult offspring mice following maternal inflammation during pregnancy. Developmental psychobiology. 2006;48(2):162–8. Epub 2006/02/21. doi: 10.1002/dev.20116 . 16489598 10.1002/dev.20116

[23] Depino AM. Early prenatal exposure to LPS results in anxiety- and depression-related behaviors in adulthood. Neuroscience. 2015;299:56–65. Epub 2015/05/07. doi: 10.1016/j.neuroscience.2015.04.065 . 25943476 10.1016/j.neuroscience.2015.04.065

[24] Lambert JF, Benoit BO, Colvin GA, Carlson J, Delville Y, Quesenberry PJ. Quick sex determination of mouse fetuses. Journal of neuroscience methods. 2000;95(2):127–32. Epub 2000/04/07. 10752483 10.1016/s0165-0270(99)00157-0

[25] Pernot F, Dorandeu F, Beaup C, Peinnequin A. Selection of reference genes for real-time quantitative reverse transcription-polymerase chain reaction in hippocampal structure in a murine model of temporal lobe epilepsy with focal seizures. Journal of neuroscience research. 2010;88(5):1000–8. Epub 2009/11/26. doi: 10.1002/jnr.22282 . 19937810 10.1002/jnr.22282

[26] Veazey KJ, Golding MC. Selection of stable reference genes for quantitative rt-PCR comparisons of mouse embryonic and extra-embryonic stem cells. PloS one. 2011;6(11):e27592 Epub 2011/11/22. doi: 10.1371/journal.pone.0027592 ; 22102912 10.1371/journal.pone.0027592 PMC3213153

[27] Vasilache AM, Kugelberg U, Blomqvist A, Nilsberth C. Minor changes in gene expression in the mouse preoptic hypothalamic region by inflammation-induced prostaglandin E2. Journal of neuroendocrinology. 2013;25(7):635–43. Epub 2013/05/02. doi: 10.1111/jne.12044 . 23631667 10.1111/jne.12044

[28] Chen YS, Lin HH, Hsueh PT, Ni WF, Liu PJ, Chen PS, et al Involvement of L-selectin expression in Burkholderia pseudomallei-infected monocytes invading the brain during murine melioidosis. Virulence. 2016:1–16. Epub 2016/09/21. doi: 10.1080/21505594.2016.1232239 . 27646437 10.1080/21505594.2016.1232239 PMC5626245

[29] Varghese F, Bukhari AB, Malhotra R, De A. IHC Profiler: an open source plugin for the quantitative evaluation and automated scoring of immunohistochemistry images of human tissue samples. PloS one. 2014;9(5):e96801 Epub 2014/05/08. doi: 10.1371/journal.pone.0096801 ; 24802416 10.1371/journal.pone.0096801 PMC4011881

[30] Sakowski SA, Geddes TJ, Thomas DM, Levi E, Hatfield JS, Kuhn DM. Differential tissue distribution of tryptophan hydroxylase isoforms 1 and 2 as revealed with monospecific antibodies. Brain research. 2006;1085(1):11–8. Epub 2006/04/04. doi: 10.1016/j.brainres.2006.02.047 . 16581041 10.1016/j.brainres.2006.02.047

[31] Rath MF, Coon SL, Amaral FG, Weller JL, Moller M, Klein DC. Melatonin Synthesis: Acetylserotonin O-Methyltransferase (ASMT) Is Strongly Expressed in a Subpopulation of Pinealocytes in the Male Rat Pineal Gland. Endocrinology. 2016;157(5):2028–40. Epub 2016/03/08. doi: 10.1210/en.2015-1888 ; 26950199 10.1210/en.2015-1888 PMC4870883

[32] Claustrat B, Leston J. Melatonin: Physiological effects in humans. Neuro-Chirurgie. 2015;61(2–3):77–84. Epub 2015/04/25. doi: 10.1016/j.neuchi.2015.03.002 . 25908646 10.1016/j.neuchi.2015.03.002

[33] Accardo JA, Malow BA. Sleep, epilepsy, and autism. Epilepsy & behavior: E&B. 2015;47:202–6. Epub 2014/12/17. doi: 10.1016/j.yebeh.2014.09.081 . 25496798 10.1016/j.yebeh.2014.09.081

[34] Simic G, Babic Leko M, Wray S, Harrington CR, Delalle I, Jovanov-Milosevic N, et al Monoaminergic neuropathology in Alzheimer's disease. Progress in neurobiology. 2017;151:101–38. Epub 2016/04/17. doi: 10.1016/j.pneurobio.2016.04.001 ; 27084356 10.1016/j.pneurobio.2016.04.001 PMC5061605

[35] Waider J, Araragi N, Gutknecht L, Lesch KP. Tryptophan hydroxylase-2 (TPH2) in disorders of cognitive control and emotion regulation: a perspective. Psychoneuroendocrinology. 2011;36(3):393–405. Epub 2011/01/25. doi: 10.1016/j.psyneuen.2010.12.012 . 21257271 10.1016/j.psyneuen.2010.12.012

[36] Vasudeva RK, Lin RC, Simpson KL, Waterhouse BD. Functional organization of the dorsal raphe efferent system with special consideration of nitrergic cell groups. Journal of chemical neuroanatomy. 2011;41(4):281–93. Epub 2011/06/07. doi: 10.1016/j.jchemneu.2011.05.008 . 21640185 10.1016/j.jchemneu.2011.05.008

[37] Abney M, McPeek MS, Ober C. Broad and narrow heritabilities of quantitative traits in a founder population. American journal of human genetics. 2001;68(5):1302–7. Epub 2001/04/20. doi: 10.1086/320112 ; 11309690 10.1086/320112 PMC1226113

[38] Li Z, Chalazonitis A, Huang YY, Mann JJ, Margolis KG, Yang QM, et al Essential roles of enteric neuronal serotonin in gastrointestinal motility and the development/survival of enteric dopaminergic neurons. The Journal of neuroscience: the official journal of the Society for Neuroscience. 2011;31(24):8998–9009. Epub 2011/06/17. doi: 10.1523/jneurosci.6684-10.2011 ; 21677183 10.1523/JNEUROSCI.6684-10.2011 PMC4442094

[39] Gentile MT, Nawa Y, Lunardi G, Florio T, Matsui H, Colucci-D'Amato L. Tryptophan hydroxylase 2 (TPH2) in a neuronal cell line: modulation by cell differentiation and NRSF/rest activity. Journal of neurochemistry. 2012;123(6):963–70. Epub 2012/09/11. doi: 10.1111/jnc.12004 . 22958208 10.1111/jnc.12004

[40] Howerton CL, Bale TL. Prenatal programing: at the intersection of maternal stress and immune activation. Hormones and behavior. 2012;62(3):237–42. Epub 2012/04/03. doi: 10.1016/j.yhbeh.2012.03.007 ; 22465455 10.1016/j.yhbeh.2012.03.007 PMC3568743

[41] Dantzer R. Cytokine, sickness behavior, and depression. Immunology and allergy clinics of North America. 2009;29(2):247–64. Epub 2009/04/25. doi: 10.1016/j.iac.2009.02.002 ; 19389580 10.1016/j.iac.2009.02.002 PMC2740752

[42] Wu WL, Hsiao EY, Yan Z, Mazmanian SK, Patterson PH. The placental interleukin-6 signaling controls fetal brain development and behavior. Brain, behavior, and immunity. 2017;62:11–23. Epub 2016/11/14. doi: 10.1016/j.bbi.2016.11.007 ; 27838335 10.1016/j.bbi.2016.11.007 PMC5373986

[43] Kubesova A, Tejkalova H, Syslova K, Kacer P, Vondrousova J, Tyls F, et al Biochemical, histopathological and morphological profiling of a rat model of early immune stimulation: relation to psychopathology. PloS one. 2015;10(1):e0115439 Epub 2015/01/21. doi: 10.1371/journal.pone.0115439 ; 25602957 10.1371/journal.pone.0115439 PMC4300081

[44] Arreola R, Alvarez-Herrera S, Perez-Sanchez G, Becerril-Villanueva E, Cruz-Fuentes C, Flores-Gutierrez EO, et al Immunomodulatory Effects Mediated by Dopamine. Journal of immunology research. 2016;2016:3160486 Epub 2016/11/01. doi: 10.1155/2016/3160486 ; 27795960 10.1155/2016/3160486 PMC5067323

[45] Money KM, Stanwood GD. Developmental origins of brain disorders: roles for dopamine. Frontiers in cellular neuroscience. 2013;7:260 Epub 2014/01/07. doi: 10.3389/fncel.2013.00260 ; 24391541 10.3389/fncel.2013.00260 PMC3867667

[46] Amrein I, Isler K, Lipp HP. Comparing adult hippocampal neurogenesis in mammalian species and orders: influence of chronological age and life history stage. The European journal of neuroscience. 2011;34(6):978–87. Epub 2011/09/21. doi: 10.1111/j.1460-9568.2011.07804.x . 21929629 10.1111/j.1460-9568.2011.07804.x

[47] Rousseau F, Aubrey KR, Supplisson S. The glycine transporter GlyT2 controls the dynamics of synaptic vesicle refilling in inhibitory spinal cord neurons. The Journal of neuroscience: the official journal of the Society for Neuroscience. 2008;28(39):9755–68. Epub 2008/09/26. doi: 10.1523/jneurosci.0509-08.2008 . 18815261 10.1523/JNEUROSCI.0509-08.2008 PMC6671229

[48] Bogdanik LP, Chapman HD, Miers KE, Serreze DV, Burgess RW. A MusD retrotransposon insertion in the mouse Slc6a5 gene causes alterations in neuromuscular junction maturation and behavioral phenotypes. PloS one. 2012;7(1):e30217 Epub 2012/01/25. doi: 10.1371/journal.pone.0030217 ; 22272310 10.1371/journal.pone.0030217 PMC3260239

[49] Martin ER, Menold MM, Wolpert CM, Bass MP, Donnelly SL, Ravan SA, et al Analysis of linkage disequilibrium in gamma-aminobutyric acid receptor subunit genes in autistic disorder. American journal of medical genetics. 2000;96(1):43–8. Epub 2000/02/25. . 10686550 10.1002/(sici)1096-8628(20000207)96:1<43::aid-ajmg9>3.0.co;2-3

[50] Khananshvili D. The SLC8 gene family of sodium-calcium exchangers (NCX)—structure, function, and regulation in health and disease. Molecular aspects of medicine. 2013;34(2–3):220–35. Epub 2013/03/20. doi: 10.1016/j.mam.2012.07.003 . 23506867 10.1016/j.mam.2012.07.003

[51] Castillo A, Kramer N, Schwartz CE, Miles JH, DuPont BR, Rosenfeld JA, et al 19q13.32 microdeletion syndrome: three new cases. European journal of medical genetics. 2014;57(11–12):654–8. Epub 2014/09/18. doi: 10.1016/j.ejmg.2014.08.009 . 25230004 10.1016/j.ejmg.2014.08.009

[52] Zager A, Peron JP, Mennecier G, Rodrigues SC, Aloia TP, Palermo-Neto J. Maternal immune activation in late gestation increases neuroinflammation and aggravates experimental autoimmune encephalomyelitis in the offspring. Brain, behavior, and immunity. 2015;43:159–71. Epub 2014/08/12. doi: 10.1016/j.bbi.2014.07.021 . 25108214 10.1016/j.bbi.2014.07.021

[53] Hong H, Kim BS, Im HI. Pathophysiological Role of Neuroinflammation in Neurodegenerative Diseases and Psychiatric Disorders. International neurourology journal. 2016;20(Suppl 1):S2–7. Epub 2016/05/28. doi: 10.5213/inj.1632604.302 ; 27230456 10.5213/inj.1632604.302 PMC4895907

[54] Sofroniew MV, Vinters HV. Astrocytes: biology and pathology. Acta neuropathologica. 2010;119(1):7–35. Epub 2009/12/17. doi: 10.1007/s00401-009-0619-8 ; 20012068 10.1007/s00401-009-0619-8 PMC2799634

[55] Kim YS, Leventhal BL, Koh YJ, Fombonne E, Laska E, Lim EC, et al Prevalence of autism spectrum disorders in a total population sample. The American journal of psychiatry. 2011;168(9):904–12. Epub 2011/05/12. doi: 10.1176/appi.ajp.2011.10101532 . 21558103 10.1176/appi.ajp.2011.10101532

[56] Krishna S, Dodd CA, Filipov NM. Behavioral and monoamine perturbations in adult male mice with chronic inflammation induced by repeated peripheral lipopolysaccharide administration. Behavioural brain research. 2016;302:279–90. Epub 2016/01/24. doi: 10.1016/j.bbr.2016.01.038 ; 26802725 10.1016/j.bbr.2016.01.038 PMC4769664

[57] Carvey PM, Chang Q, Lipton JW, Ling Z. Prenatal exposure to the bacteriotoxin lipopolysaccharide leads to long-term losses of dopamine neurons in offspring: a potential, new model of Parkinson's disease. Frontiers in bioscience: a journal and virtual library. 2003;8:s826–37. Epub 2003/09/06. . 12957870 10.2741/1158

[58] Altieri SC, Yang H, O'Brien HJ, Redwine HM, Senturk D, Hensler JG, et al Perinatal vs genetic programming of serotonin states associated with anxiety. Neuropsychopharmacology: official publication of the American College of Neuropsychopharmacology. 2015;40(6):1456–70. Epub 2014/12/20. doi: 10.1038/npp.2014.331 ; 25523893 10.1038/npp.2014.331 PMC4397404

[59] Chakravarty S, Herkenham M. Toll-like receptor 4 on nonhematopoietic cells sustains CNS inflammation during endotoxemia, independent of systemic cytokines. The Journal of neuroscience: the official journal of the Society for Neuroscience. 2005;25(7):1788–96. Epub 2005/02/18. doi: 10.1523/jneurosci.4268-04.2005 . 15716415 10.1523/JNEUROSCI.4268-04.2005 PMC6725921

[60] Csordas T, Bertok L, Csapo Z. Experiments on prevention of the endotoxin-abortifacient effect by radiodetoxified endotoxin pretreatment in rats. Gynecologic and obstetric investigation. 1978;9(1):57–64. Epub 1978/01/01. . 361514 10.1159/000300971

[61] Dutkiewicz J, Mackiewicz B, Lemieszek MK, Golec M, Milanowski J. Pantoea agglomerans: a marvelous bacterium of evil and good. Part I. Deleterious effects: Dust-borne endotoxins and allergens—focus on cotton dust. Annals of agricultural and environmental medicine: AAEM. 2015;22(4):576–88. Epub 2015/12/29. doi: 10.5604/12321966.1185757 . 26706959 10.5604/12321966.1185757

[62] Estes ML, McAllister AK. Maternal immune activation: Implications for neuropsychiatric disorders. Science (New York, NY). 2016;353(6301):772–7. Epub 2016/08/20. doi: 10.1126/science.aag3194 . 27540164 10.1126/science.aag3194 PMC5650490

[63] Careaga M, Murai T, Bauman MD. Maternal Immune Activation and Autism Spectrum Disorder: From Rodents to Nonhuman and Human Primates. Biological psychiatry. 2017;81(5):391–401. Epub 2017/02/01. doi: 10.1016/j.biopsych.2016.10.020 . 28137374 10.1016/j.biopsych.2016.10.020 PMC5513502

[64] Altemus M, Sarvaiya N, Neill Epperson C. Sex differences in anxiety and depression clinical perspectives. Frontiers in neuroendocrinology. 2014;35(3):320–30. Epub 2014/06/03. doi: 10.1016/j.yfrne.2014.05.004 ; 24887405 10.1016/j.yfrne.2014.05.004 PMC4890708

[65] Liu B, Xie JX, Rowlands DK, Gou YL, Leung CC, Chung YW, et al Neuroprotective effects of Bak Foong Pill in 1-methyl-4-phenyl-1,2,3,6-tetrahyrdropyridine (MPTP)-induced Parkinson's disease model mice. Biological & pharmaceutical bulletin. 2004;27(8):1245–50. Epub 2004/08/12. . 15305030 10.1248/bpb.27.1245

