# Supplementary figures and images for: Expression of cerebral serotonin related to anxiety-like behaviors in C57BL/6 offspring induced by repeated subcutaneous prenatal exposure to low-dose lipopolysaccharide
Source: PLoS One. 2017 Jun 26;12(6):e0179970. doi: 10.1371/journal.pone.0179970 (PMC5484498; doi:10.1371/journal.pone.0179970)

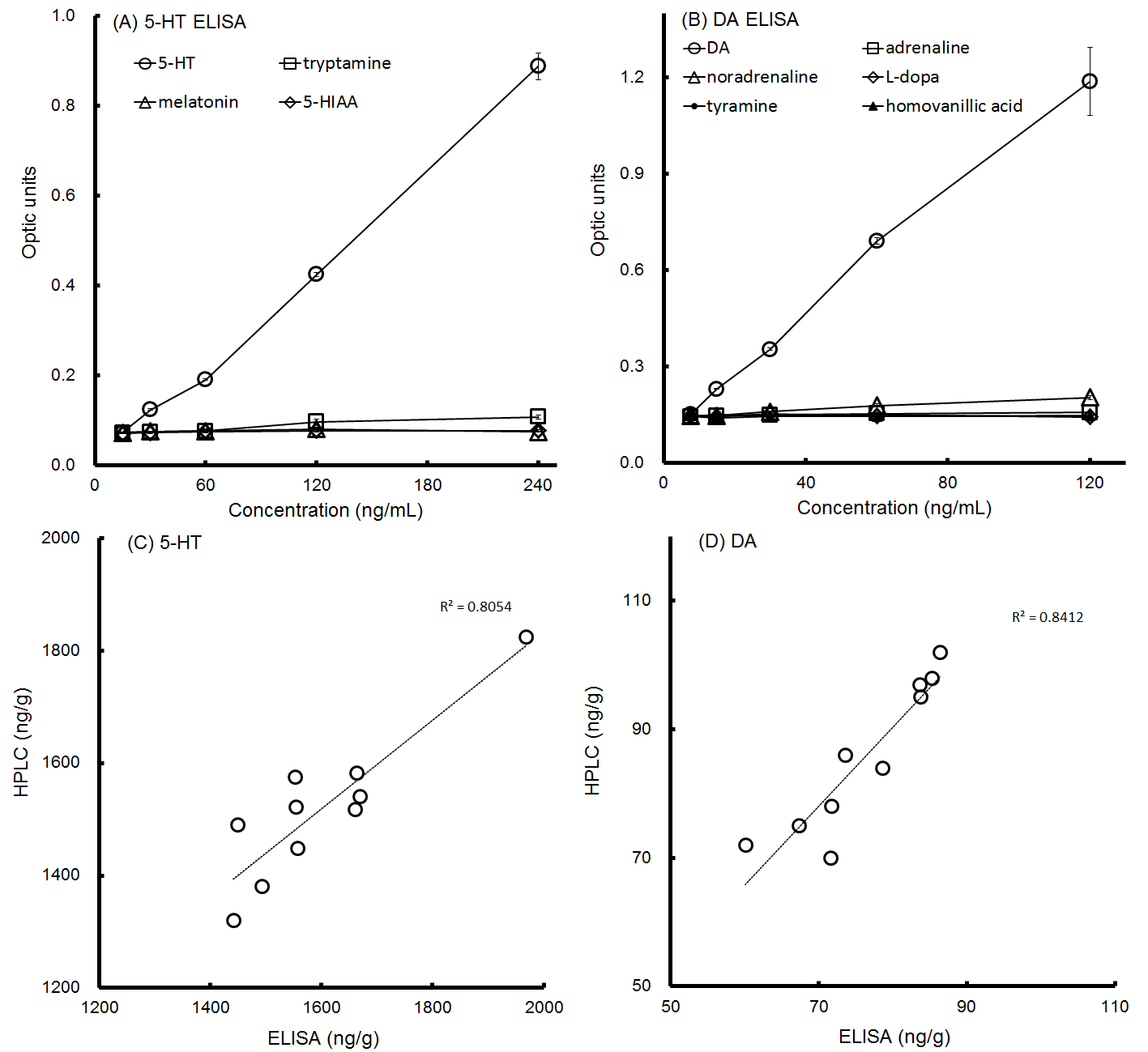

Supplement: S1 Fig — The brain tissues (containing the intact cerebral cortex, hippocampus, substantia nigra, midbrain, hind brain and brain stem) were weighed, homogenized and deproteinized in 300 μL of a 0.2 N perchloric acid solution. After centrifugation (14,000 x g for 30 min at 4°C), the supernatants were filtered through 0.2 μm membrane and analyzed using high pressure liquid chromatography (5 μL; HPLC, Hitachi Chromaster System, Petrzikova, Praha, Czech Republic) equipped with a reverse-phase column (Thermo Scientific Syncronis C18, 5.0 μm, 4.6 x 250 mm; Fisher Scientific Co., PA, USA), colorimetric detector (L-2400, Hitachi Co.), and 6011RS ultra analytical cell and pump (L-2130, Hitachi Co.). The mobile phase was 75 mM Na2HPO4, 1.7 mM 1-octanesulfonic acid, 100 μL/L triethylamine, 25 μM ethylenediaminetetraacetic acid and 10% (v/v) acetonitrile (pH 3.0), and the flow rate was 0.5 mL/min. The concentration of both 5-HT and DA was determined using the areas of the sample peaks against the areas of the reference 5-HT and DA standards (Sigma Co.) at a specific retention time (5-HT at 6.38 min; DA at 13.28 min). For the ELISA, the brain tissues were prepared in 300 μL of PBS (see Materials and methods). The OD values of 5-HT, 5-hydroxyindoleacetic acid (5-HIAA), melatonin and tryptamine, at concentrations ranging from 15 ng/mL to 240 ng/mL, as measured by ELISA at 450 nm, are shown (A). The OD values of DA, adrenaline, noradrenaline, L-dopa, tyramine and homovanillic acid, at concentrations ranging from 15 ng/mL to 120 ng/mL, as measured by ELISA at 450 nm, are shown (B). A total of 10 representative brain tissues (n = 5, LPS-induced brains; n = 5, PBS-treated controls) were homogenized with 300 μL PBS and separated into two equal volumes (150 μL for each). One was diluted into 300 μL PBS for ELISA and another was diluted into a final concentration of 0.2 N perchloric acid solutions (300 μL) for HPLC. The concentration (ng/g) of the neurotransmitter each paired sample was respectiv [file pone.0179970.s001.tif]
