# Supplementary material for: Expression of cerebral serotonin related to anxiety-like behaviors in C57BL/6 offspring induced by repeated subcutaneous prenatal exposure to low-dose lipopolysaccharide
Source: PLoS One. 2017 Jun 26;12(6):e0179970. doi: 10.1371/journal.pone.0179970 (PMC5484498; doi:10.1371/journal.pone.0179970)
Supplement: S1 Table — (DOCX) [file pone.0179970.s002.docx]

Table S1 Dose effects of LPS on the abortion and survival of offspring after maternal immune activation

| Pregnant mice | LPS (μg/Kg) injection at | | | Abortions | Births | Survival (%) |
| --- | --- | --- | --- | --- | --- | --- |
| (number) | GD15^a^ | GD16 | GD17 | (%) | (number) | at 5 weeks old |
| 5 | 60 | 60 | 80 | 60% (3/5) | 16 | 18.7% (3/16) |
| 3 | 50 | 50 | 50 | 66% (2/3) | 7 | 42.8% (3/7) |
| 9 | 25 | 25 | 50 | 11% (1/9) | 64 | 92% (59/64) |

^a^ gestational day
